# Perched on the Plateau: Speciation in a Cape Fold Mountain Velvet Worm Clade, With the Description of Seven New Species (Onychophora: Peripatopsidae: *Peripatopsis*) From South Africa

**DOI:** 10.1002/ece3.71256

**Published:** 2025-04-20

**Authors:** Savel R. Daniels, Aaron Barnes

**Affiliations:** ^1^ Department of Botany and Zoology Stellenbosch University Matieland South Africa

**Keywords:** allopatric diversification, biodiversity, invertebrate taxonomy, novel lineages

## Abstract

During the present study, we re‐examined species boundaries in three mountain‐dwelling velvet worm species complexes (*Peripatopsis balfouri s.l., P. bolandi s.l.* and *P. purpureus s.l*.) along the Cape Fold Mountains of South Africa. We obtained DNA sequence data for both the mitochondrial cytochrome *c* oxidase subunit one (*COI*) and the nuclear *18S rRNA* loci. Phylogenetic inferences were derived with the use of maximum likelihood and Bayesian inference coupled with a divergence time estimation. Four species delimitation methods (ASAP, bPTP, bGYMC and STACEY) together with gross morphological analyses and scanning electron microscopy (SEM) were used to validate the diagnosis of novel species. Combined phylogenetic results demonstrated the presence of three geographically discrete clades (A‐C). Corroborative evidence for the novel lineages could be derived from the dorsal integument colour of live specimens and fixed dorsal and ventral papilla scales rank counts. The four species delimitation methods produced variable results. Divergence time estimations indicated that the Miocene epochs was a major period of cladogenesis. The seven novel velvet worm species, *P. barnardi* **sp. nov**., *P. fernkloofi* **sp. nov**., *P. jonkershoeki* **sp. nov**., *P. kogelbergi* **sp. nov**., *P. landroskoppie* **sp. nov**., *P. limietbergi* **sp. nov**., and *P. palmeri* **sp. nov**., are herein described.

## Graphical abstract

Molecular systematic studies revealed seven novel velvet worm species from the Cape Fold Mountains in the Western Cape, South Africa suggest high levels of endemism for forest‐dwelling species such a velvet worms (*Peripatopsis*). Photographic images of five of the new species (B‐F).

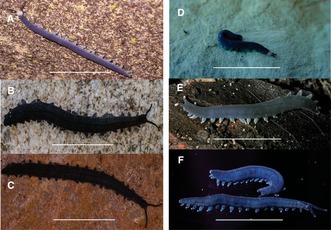

## 1 Introduction

The Cape Fold Mountains (CFM) situated in a global biodiversity hotspot in the Western Cape province of South Africa is Palaeozoic in orogenesis and has undergone dramatic erosion since (Cowling et al. 2009). The CFM runs in a north–south‐east axis parallel to the coast and adjacent interior where it is comprised of several disjunct, geographically discrete mountain blocks (Cowling et al. 2009). Along the CFM belt, fragmented Afrotemperate forest patches persist in deep gorges (Mucina and Rutherford 2011). The antiquity and paleo‐distribution of the Afrotemperate forests in the Western Cape province are disputed and a matter of considerable debate (Mucina and Rutherford 2011; McDonald and Daniels 2012). During the early Miocene, the region was mesic and subtropical; however, during the late Miocene, with the advent of the proto‐Benguela current along the west coast (Siesser 1980), the region underwent significant climatic deterioration marked by decreases in rainfall and the subsequent onset of xeric climatic regimes and became more temperate (Cowling et al. 2009; Mucina and Rutherford 2011). The aridification cycles continued and intensified during the Plio/Pleistocene. Periodic climatic shifts from xeric to mesic resulted in a complex mosaic of habitat connectivity and isolation. These Mio/Plio/Pleistocene climate shifts in concert with orographic uplift events, landscape erosion cycles, and marine transgressions are thought to have been a catalyst in the speciation of faunal lineages (Engelbrecht et al. 2013; Diedericks and Daniels 2014; Hofmeyr et al. 2020; Barnes et al. 2020; Myburgh and Daniels 2022). Ancient habitat specialists euarthropod lineages, such as velvet worms (Onychophora), inhabiting the Afrotemperate forests distributed along the CFM belt may provide valuable insight into the drivers of cladogenesis (McDonald and Daniels 2012; Barnes and Daniels 2022). Consequently, velvet worms are ideal organisms with which to explore the impact of spatiotemporal disjunction induced by climatic ameliorations (Daniels et al. 2017; Giribet et al. 2018; McDonald and Daniels 2012; Murienne et al. 2014; Myburgh and Daniels 2022; Trewick et al. 2024).

Two velvet worm genera are present in South Africa, *Peripatopsis* (Pocock 1894) and *Opisthopatus* (Purcell 1899) containing seven and three species respectively (Hamer et al. 1997; Ruhberg and Hamer 2005). Traditionally, species boundaries were defined based on dubious taxonomic characters such as, for example, leg pair numbers and colour, resulting in an unreliable estimation of the number of species (Daniels et al. 2009). Evolutionary studies of *Peripatopsis* using modern systematic methods have culminated in the discovery and description of 20 new species (McDonald et al. 2012; Daniels et al. 2013; Ruhberg and Daniels 2013; Barnes et al. 2020; Grobler et al. 2023; Nieto Lawrence and Daniels 2024; Barnes and Daniels 2024). Most recently described *Peripatopsis* species are point endemic, suggesting fine‐scale sampling is critical to detect alpha taxonomic diversity (Ruhberg and Daniels 2013; Grobler et al. 2023; Nieto Lawrence and Daniels 2024). Despite continuous recent sampling efforts, large geographic areas of South Africa and, particularly, in the steep valleys and deep gorges along the coastal margins and adjacent interior of the heterogeneous CFM of the Western Cape province remain unsampled.

Citizen science platforms can form a crucial link in documenting biodiversity in unsampled areas for poorly studied faunal groups such as velvet worms (Daniels et al. 2022). Recently, two curious posts to the citizen science platform iNaturalist sparked renewed interest in the *Peripatopsis* diversity of the Western Cape province, South Africa. The first iNaturalist post, a video together with photographic images, revealed a pearl‐white velvet worm crawling on boulders under water in a streambed at Porterville along the Groot Winterhoek Mountains. A second iNaturalist post showed a velvet worm from the Groot Swartberg Mountains in the Little Karoo, a semi‐arid region in the interior of the Western Cape province from where no velvet worm records exist (Hamer et al. 1997). Both iNaturalist velvet worm posts are morphologically part of the *P. balfouri s*.*l*. Sedgwicki, 1885 species complex, reigniting interest into species boundaries in the *P. balfouri s.l*. species complex, where, following reinterrogation of published data (Daniels et al. 2009, 2013), it became apparent that species diversity was underestimated in the initial study by Daniels et al. (2013). The latter authors evaluated species boundaries in the *P. balfouri s*.*l*. complex and described three new species. However, considering the deep divergence evident from the mitochondrial cytochrome *c* oxidase one locus (*COI*), fixed nuclear differences in the *18S rRNA* locus, some additional novel species likely exist in *P. balfouri s.s*., *P. bolandi s.s*. Daniels et al. 2013, and *P. purpureus s.s*. Daniels et al. 2013 (Daniels pers. obs.). During the present study, we refine species boundaries within the latter three species complexes by reinterrogating the existing data in combination with new mitochondrial and nuclear DNA sequence, gross morphological, and SEM data. In all three species complexes, allopatric clades that are highly divergent are present.

The present study has two objectives. First, to determine the phylogenetic placement of the velvet worm specimens posted to iNaturalist. Second, to refine species in the three (*P. balfouri s.l*., *P. bolandi s.l*. and *P. purpureus s.l*.) species complexes using phylogenetic inference, species delimitation methods, gross morphological examination, and SEM. We hypothesise that the specimens from the two iNaturalist records represent novel species and that species diversity in the three aforementioned species complexes was underestimated. We address these objectives by undertaking renewed sampling of velvet worms in the CFM in combination with mitochondrial and nuclear DNA sequencing from two preexisting studies (Daniels et al. 2009, 2013).

## 2 Materials and Methods

**FIGURE 1:**
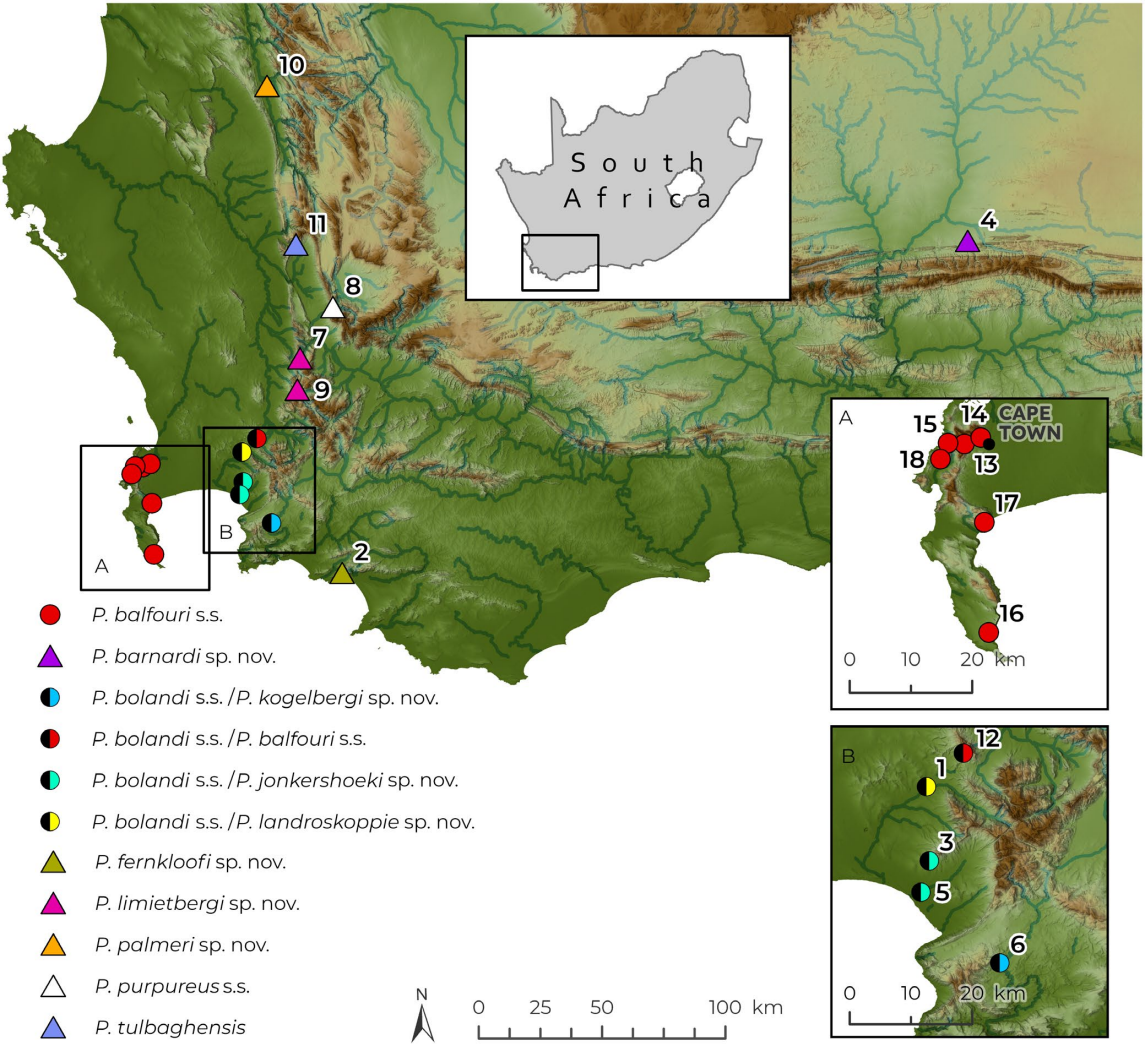
Map of sample localities where velvet worms (*Peripatopsis*) were collected in the Western Cape province, South Africa. The numbers (1–18) on the map correspond to the sample localities in Table 1.

During the present study, we collected velvet worm specimens from Porterville in the Groot Winterhoek and from the Groot Swartberg Mountains, Little Karoo, Western Cape province, South Africa, based on the iNaturalist records. In addition, we recollected specimens from Du Toitskloof (Mollenaars Hike) for *P. purpureus s.s*., and finally, we resampled specimens from Helderberg, Jonkershoek, and Kogelberg Biosphere Nature Reserves for *P. bolandi s.s*. (Table 1; Figure 1). We also included two localities of *P. cederbergiensis* Daniels et al. 2013 (Boschkloof and Helskloof in the Cederberg Mountains) and one of *P. alba* (Lawrence 1931) from the Wynberg Cave, Cape Peninsula. Velvet worms were hand collected from under logs or stones, under moss close to stream beds, or inside decomposing leaf litter and placed into a labelled plastic jars. The coordinates of the locality from where velvet worms were sampled were recorded with the use of a handheld GPS device (Garmin). Selected live specimens were photographed in the field using a digital camera (see morphological section). Following the capture of photographic images, velvet worms were preserved directly in 96% ethanol for molecular research.

**TABLE 1:** List of localities where velvet worms were collected from during the present study, combined with specimens collected during two previous studies (Daniels et al. 2009, 2013).

Locality	Locality	Species	S	E	Previous study	Present study	Total
1	Landroskop A/B	*P. bolandi s.s./P. landroskoppie* **sp. nov**.	33°56′ 23′′	18°51′ 20′′	5/1'		5/1'
2	Fernkloof Nature Reserve	*P. fernkloofi* **sp. nov**.	34°23′ 37′′	19°16′ 34′′	3		3
3	Helderberg Nature Reserve	*P. bolandi s.s./P. jonkershoeki* **sp. nov**.	34°03′ 00′′	18°51′ 00′′	8	7	8/7'
4	Groot Swartberg Mountains, Little Karoo	*P. barnardi* **sp. nov**.	33°13′ 34′′	22°01′ 18′′		4	4
5	Kogelberg Biosphere Nature Reserve A/B	*P. bolandi s.s*.*/P. kogelbergi* **sp. nov**.	34°05′ 46′′	18°50′ 30′′	3/2'	4	7/2'
6	Jonkershoek Nature Reserve A/B	*P. bolandi s.s*.*/P. jonkershoeki* **sp. nov**.	34°12′ 20′′	18°58′ 14′′	4/6'	2	4/8'
7	Bainskloof Pass	*P. limietbergi* **sp. nov**.	33°35′ 48′′	19°07′ 12′′	1		1
8	Mitchell's Pass	*P. limietbergi* **sp. nov**.	33°24′ 44′′	19°16′ 30′′	5		5
9	Du Toitskloof Pass	*P. purpureus s.s*.	33°42′ 43′′	19°06′ 33′′	4	3	7
10	Porterville, Groot Winterhoek Mountains	*P. palmeri* **sp. nov**.	32°59′ 24′′	19°01′ 30′′		6	6
11	Tulbagh	*P. tulbaghensis*	33°10′ 39′′	19°07′ 56′′	7		7
12	Simonsberg A/B	*P. bolandi s.s./P. balfouri s.s*.	33°53′ 31′′	18°55′ 25′′	1/7'		1/7'
13	Kirstenbosch	*P. balfouri s.s*.	33°58′ 48′′	18°25′ 48′′	1		1
14	Newlands Forest	*P. balfouri s.s*.	33°58′ 12′′	18°27′ 00′′	4/4'		8
15	Slangolie	*P. balfouri s.s*.	33°58′ 39′′	18°23′ 00′′	3		3
16	Booi se Skerm	*P. balfouri s.s*.	34°18′ 16′′	18°27′ 36′′	5		5
17	St James	*P. balfouri s.s*.	34°06′ 56′′	18°27′ 30′′	3		3
18	Orange Kloof	*P. balfouri s.s*.	34°00′ 16′′	18°22′ 59′′	3		3

*Note:* The locality numbers correspond to those on the map (Figure 1).

### 2.1 DNA Sequencing

A small tissue biopsy (2–3 mm) was taken per specimen and subjected to DNA extraction using the Machery‐Nagel DNA extraction kit, following the manufacturer's protocol. The DNA was stored in a refrigerator at 4°C until required for the polymerase chain reaction (PCR). The DNA extractions were diluted after extraction with 1 μL of DNA in 19 μL of water. Two gene fragments were amplified and sequenced. These include the mitochondrial (mtDNA) *COI* locus and the nuclear *18S rRNA* subunits (*18S rRNA*). The primer pairs for *COI* were obtained from Folmer et al. (1994), while the *18S rRNA* primer pairs, 5F and 7R, were obtained from Giribet et al. (1996). Both loci have been extensively used in velvet worm evolutionary studies (Barnes and Daniels 2019, 2022, 2024; Barnes et al. 2020; Daniels et al. 2009, 2013, 2016, 2017; Giribet et al. 2018; McDonald and Daniels 2012; Murienne et al. 2014; Myburgh and Daniels 2015; Sato et al. 2018; Grobler et al. 2023; Lord et al. 2024). All specimens were sequenced for *COI*, while a single representative per locality was sequenced for the *18S rRNA* locus except where sympatric lineages were present at a sample locality. Standard PCR conditions for *COI* and *18S rRNA* were followed as outlined by Grobler et al. (2023) and Nieto Lawrence and Daniels (2024). The PCR products were electrophoresed in a 1% agarose gel for 180 min at 90 V, and a Bio flux gel purification kit was used to clean the gel‐excised PCR products. Sequencing was conducted with the use of an ABI 3730 machine at the DNA sequence facility of the University of Stellenbosch.

### 2.2 Phylogenetic Analyses

To check for base ambiguities and compute a consensus sequence, forward and reverse strands were used through the use of SEQUENCE NAVIGATOR (Applied Biosystems, Foster City, CA, USA). CLUSTAL (ver. University College of Dublin, Dublin, Ireland, see http://www.clustal.org, accessed August 2024; Thompson and Plewniak 1997). The DNA sequences of sister species were obtained from Daniels et al. (2009, 2013) and Barnes et al. (2020). Two velvet worm species, *P. capensis* and *P. overbergiensis* (McDonald et al. 2012) were selected as outgroups based Daniels et al. (2009). The DNA sequence data from the present study was combined with data for *P. alba* , *P. balfouri s.s*., *P. bolandi s.s*. and *P. purpureus s.s*., from two previous studies (Daniels et al. 2009, 2013) (Figure 1; Table 1). Furthermore, phylogenetic studies on the latter group revealed a close evolutionary relationship between the southern Cape species, *P. clavigera* (Purcell 1899), *P. edenensis* (Barnes et al. 2020), *P. ferox* (Barnes et al. 2020) and the two north western CFM species from the Cedarberg Mountains species *P. cederbergiensis* and *P. tulbaghensis* (Barnes et al. 2020) from Tulbagh. Hence representative DNA sequences of these five velvet worm species were included in the present study (Barnes et al. 2020; Daniels et al. 2013). Bayesian inference (BI) and maximum likelihood (ML) were used to construct the phylogeny, ML in RAxML (ver. 7.0.4, The Exelixis Lab, Heidelberg Institute for Theoretical Studies, Heidelberg, Germany; Stamatakis et al. 2008) and BI in MrBayes (ver. 3.2.2, Ronquist et al. 2012). We used the same approach for both ML and BI as outlined by Barnes and Daniels (2022). Uncorrected ‘*p*’ distances for both the *COI* and *18S rRNA* loci were calculated in PAUP*4 (Swofford 2002).

### 2.3 Divergence Time Estimate

For the *COI* locus, a divergence time estimate was conducted with the use of a Bayesian framework. This in turn uses a probability model to describe the molecular sequence of divergence of lineages. This then makes use of the Markov Chain Monte Carlo (MCMC) method to estimate the age of clades. All specimens included in the present study were combined with the *COI* data from the two previous studies for the divergence time estimation (Daniels and Ruhberg 2010; McDonald and Daniels 2012). A strict molecular clock was implemented, and the analyses were run through the use of the program BEAST2 (ver. 2.4.8, Drummond and Rambaut 2007). For the *COI l*ocus, 1.5%–2.3% substitution rates per million years were used, with a mean substitution rate of 1.9% per million years being selected (Brower 1994; Trewick and Wallis 2001; Boyer et al. 2007). A multiple coalescent model was used (Heled and Drummond 2010). jModelTest2 (ver. 2.1.6, see http://www.phylo.org/index.php/tools/jmodeltest2_ xsede.html, accessed August 2024; Posada 2000) on XSEDE through CIPRES (Miller et al. 2010) was used to assess the substitution model and parameters for the complete *COI* subset. We used the same approach for the divergence time estimate as outlined by Barnes and Daniels (2022).

### 2.4 Species Delimitation Using ASAP, bPTP, bGMYC, and STACEY

The following species delimitation methods were employed under two separate datasets for best accuracy, based on the respective methodological requirements. The first two methods, assemble species by automatic partitioning model (ASAP) and the Bayesian implementation of the Poison tree process (bPTP), are designed for single‐locus datasets; hence, the full *COI* dataset of all specimens was used. Then for both the Bayesian implementation of the GMYC (bGMYC) model and Species Tree and Classification Estimation, Yarely (STACEY), a concatenated dataset comprising the *COI* and *18S rRNA* loci (mt + nuDNA) was used with alterations due to the minimum of 50 taxa requirement for the bGMYC analysis. The singularly generated *18S rRNA* sequences from each locality included in the study, selected through preliminary *COI* analyses and accommodating for sympatry, were duplicated to accommodate for numbers under the understanding that the *18S rRNA* locus is highly conserved and unlikely to show base pair differences at the intraspecific level. The first duplicates were then paired with their corresponding *COI* sequences, followed by the inclusion of additional, as closely related *COI* sequences as possible to correspond with the second duplicates, generating a dataset of 60 taxa. This dataset was used for both bGMYC and STACEY for consistency between methods.

The first species delimitation method employed comprised the newly developed ASAP. ASAP uses genetic distances to hierarchically cluster species partitions (https:bioinfo.mnhn.fr/abi/public/asap) by first assigning a probability that each new clustering represents a new species and then computing the relative width of the barcode gap of a partition in relation to the previous partitions. These metrics are then combined into an ASAP score to rank all partitions detected in the analyses. Since ASAP is an exploratory method that does not consider the evolutionary history among sequences, we report the first two partitions ranked by ASAP score, using ‘*p*’ distances and the default setting splitting groups below a probability of < 0.01.

Second, bPTP was run on the online bPTP web server (https://species.h‐its.org/ptp/) for its ability to delimit species without a priori knowledge of population parameters (Zhang et al. 2013). A maximum likelihood tree constructed from the *COI* dataset was used for the analysis, which was run for 500,000 MCMC generations with a thinning value of 100 and burn‐in of 20%. The convergence of the MCMC chain was visually confirmed, as recommended by Zhang et al. (2013). Third, we employed a bGMYC model on a combined mt + nuDNA dataset using the R package “bGMYC” (Reid and Carstens 2012). All gene alignments were run in BEAST2 under a HKY nucleotide substitution model using a strict molecular clock. To account for error in phylogenetic estimation, 500 post‐burn‐in trees were randomly selected for analysis. A Markov chain was run for 50,000 generations, sampling the chain every 1000th generation, with 4000 generations discarded as burn‐in. A uniform prior for the number of species was applied, with a lower bound of three (two outgroup taxa and the ingroup) and an upper bound of 60 (the total number of terminals in the analysis). Convergence was assessed visually by examining the performance of the chain. The ‘check rates’ function was used to determine the rate of branching of the coalescent model to that of the Yule model.

Lastly, we employed a multilocus coalescent model using STACEY (ver. 1.2.1, http://www. beast2.org/, Jones et al. 2015) in BEAST2 for its demonstrated effectiveness in species boundaries validation (Busschau et al. 2019; Klimov et al. 2019; Prebus 2021; Williams et al. 2022). The estimated number of putative species in STACEY ranges from one to the number of putative clusters specified. Each locality was defined as a taxon set or minimal cluster, without a prior species definition and accommodating for sympatry. The input files (.xml) were created using BEAUti, implementing a mixed Yule model before estimating the species tree using the following prior settings: Collapse Height = 0.0001, Collapse Weight = 0.5 using a beta prior (1.1) around [0.1], bdcGrowthRate = log normal (M = 4.6, S = 1.5); pop‐PriorScale = log normal (M = −7, S = 2); relativeDeathRate = beta (alpha = 1.0, beta = 1.0) and strict model to describe the molecular clock. For the species‐delimitation analysis, we set the ploidy level for the *COI* locus to 0.5 and for the *18S rRNA* locus to 2.0 to account for varied substitution rates between these loci. The MCMC analysis was run for 100 million generations, saving the result every 1000 generations. The obtained log files were analysed with Tracer to verify the convergence (ESS > 200) of the analysis, and SpeciesDelimitationAnalyser (http://indriid.com/software.html, Jones et al. 2015) was used to process the log files and to examine the clusters of species assignments. Posterior probabilities of localities belonging to the same cluster were visualised in a similarity matrix constructed in R Studio (ver. 2023.09.1 + 494, RStudio: Integrated Development for R Studio Inc., Boston, MA. http://www.R‐project.org/, R Studio Team 2015).

### 2.5 Gross Morphology and Scanning Electron Microscopy (SEM)

A Canon EOS 90D camera was used to photograph the specimens, noting gross morphological traits, dorsal and ventral integument colour. A Leica microscope was used to count the number of leg pairs and note the presence of a claw on the posterior leg pair, while a digital calliper was used to measure the total length of the ethanol‐preserved specimen in millimetres (mm) from the anterior‐most point to the posterior side. The South African Museum of Cape Town has an environmental SEM, Hitachi TM4000Plus, that allows for the analysis of specimens without requiring critical point drying or sputter coating. This allowed us to preserve our limited number of specimens for use as holotypes and/or paratypes. Through the use of this SEM technique, dorsal and ventral dermal scale ranks for representatives of each of the two main clades were examined. Specimens were accessioned into the entomology collection of the South African Museum of Cape Town (SAM‐ENW‐C). All new species names were registered in ZooBank.

## 3 Results

### 3.1 Phylogenetic Inference Based on the COI Locus

**FIGURE 2:**
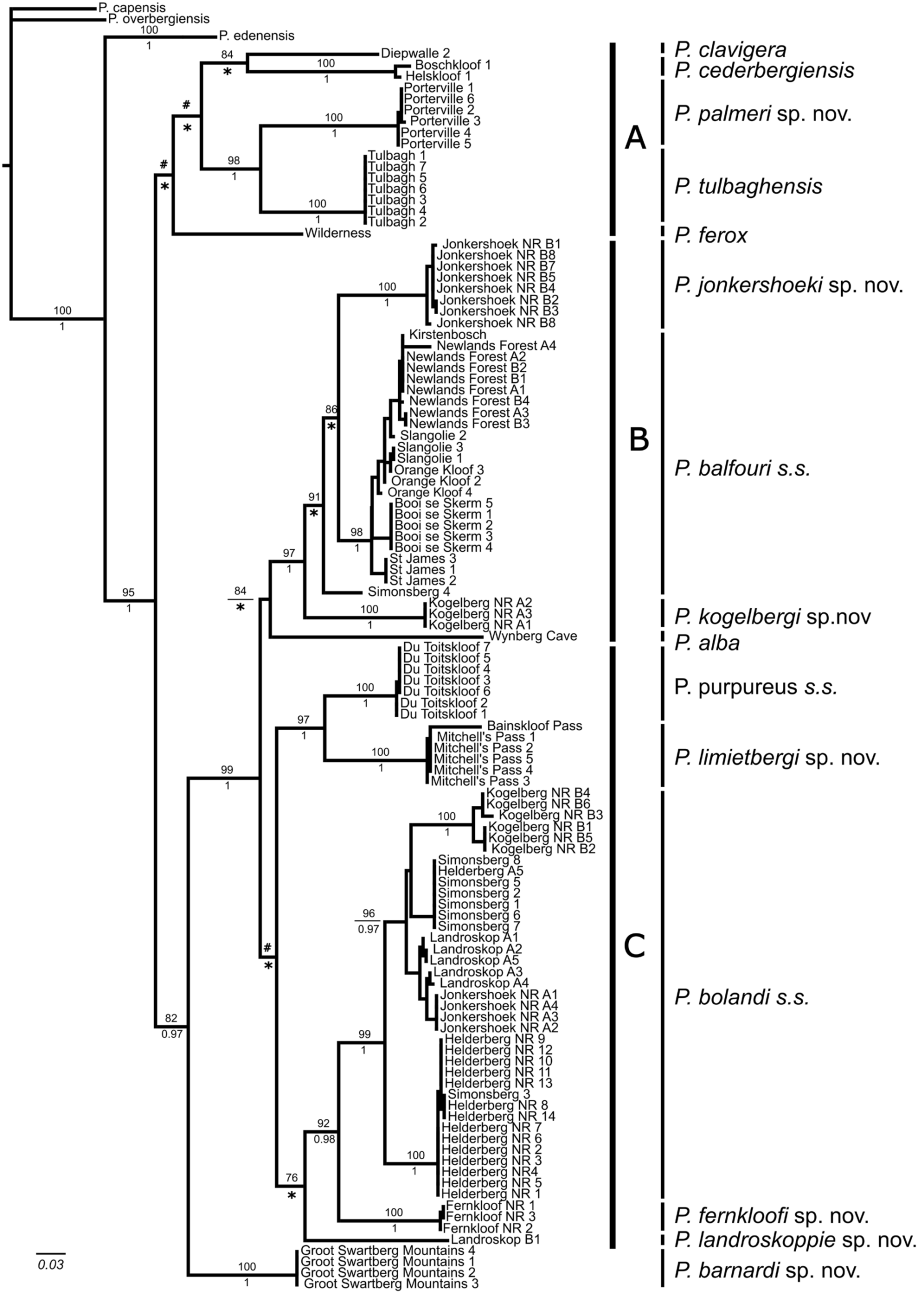
A maximum likelihood (ML) tree topology derived from the mitochondrial cytochrome oxidase c subunit one (*COI*) data showing three clades (A–C). Clade A represents southwestern Cape Fold Mountain (CFM) species, clade B represents Cape Peninsula and adjacent interior, and finally clade C represents Hottentots Holland, Kogelberg, Limietberg, and Du Toitskloof Mountains. Bootstrap values (%) for the ML analyses are provided above the node. Only bootstrap values > 75% are shown. Below the node are the posterior probability (p*P*) values derived from the Bayesian analyses. Only p*P* values > 0.95 are shown.

A 607 base pair (bp) fragment of the *COI* locus was amplified for a total of 26 new specimens. The novel *COI* sequences were deposited in GenBank (Accession numbers PP 550029–PP 550054). The newly generated *COI* data was combined with the existing *COI* dataset from the two previous studies. Phylogenetic analyses using maximum likelihood (ML) and Bayesian inference (BI) revealed identical tree topologies; hence, only the ML tree topology is shown and discussed (Figure 2). Three geographically discrete clades (A, B and C) were retrieved. In clade A, the predominantly southwestern Cape species, *P. ferox* from the Wilderness, were placed as the sister group to a clade comprised of *P. tulbaghensis* , the sister group to *P. palmeri* **sp. nov**., from Porterville in the Groot Winterhoek Mountains, while the latter clade was the sister group to a clade comprised of *P. clavigera* , the sister group to *P. cederbergiensis*. The velvet worm specimens from the Groot Swartberg Mountains in the Little Karoo, *P. barnardi* **sp. nov**., were basal to the western CFM and Cape Peninsular sister clades B and C, respectively. In clade B, *P. alba* from the Wynberg Cave on the Cape Peninsula was sister to *P. kogelbergi* **sp. nov**.; the latter species was the sister group to *P. balfouri s.s*. from the Cape Peninsula on Table Mountain (St James, Booi se Skerm, Orange Kloof, Slangolie, Newlands and Kirstenbosch) and adjacent interior (one specimen from Simonsberg Mountain) sister group to *P. jonkershoeki* **sp. nov**., from the Jonkershoek Mountains. In clade C, comprised of the Hottentots Holland Mountains and adjacent plateau species, *P. landroskoppie* **sp. nov**., was basal to *P. fernkloofi* **sp. nov**., the sister group to *P. bolandi s.s*., which included specimens from Helderberg Nature Reserve (Helderberg Mountains), Jonkerskoek A, Landroskop A, Simonsberg Mountains, and Kogelberg Biosphere Nature Reserve A. The latter clade was the sister to *P. purpureus s.s*. from Du Toitskloof and the sister group to specimens from the Limietberg at Bainskloof Pass and Mitchell's Pass, comprising *P. limietbergi* **sp. nov**.

Uncorrected ‘*p*’ sequence distance values using the *COI* locus between sister species groups was > 6%. For example, between *P. purpureus s.s*. and its sister group *P*. *limietbergi* **sp. nov**., the uncorrected distance was 7.74%. Similarly, between *P. palmeri* **sp. nov**., and its sister group *P. tulbaghensis* the uncorrected distance was 9.22%, while between *P. landroskoppie* **sp. nov**., and *P. fernkloofi* **sp. nov**., the uncorrected distance was 10.37%. Between *P. fernkloofi* **sp. nov**., and *P. bolandi s.s*. the uncorrected sequence distance was 8.07%. In the sympatric *P. bolandi s.s*. and *P. landroskoppie* **sp. nov**., at Landroskop the uncorrected distance was 13.18%. Finally, between *P. balfouri s.s*. and *P. jonkershoeki* **sp. nov**., the distance was 6.26%, and between *P. kogelbergi* **sp. nov**., and *P. jonkershoeki* **sp. nov**., the distance was 8.73%. Interspecific distances between described species, such as, for example, *P. clavigera* and *P. cederbergiensis* the uncorrected *COI* distance was 9.58%.

### 3.2 Combined COI and 18S rRNA Tree Topology

**FIGURE 3:**
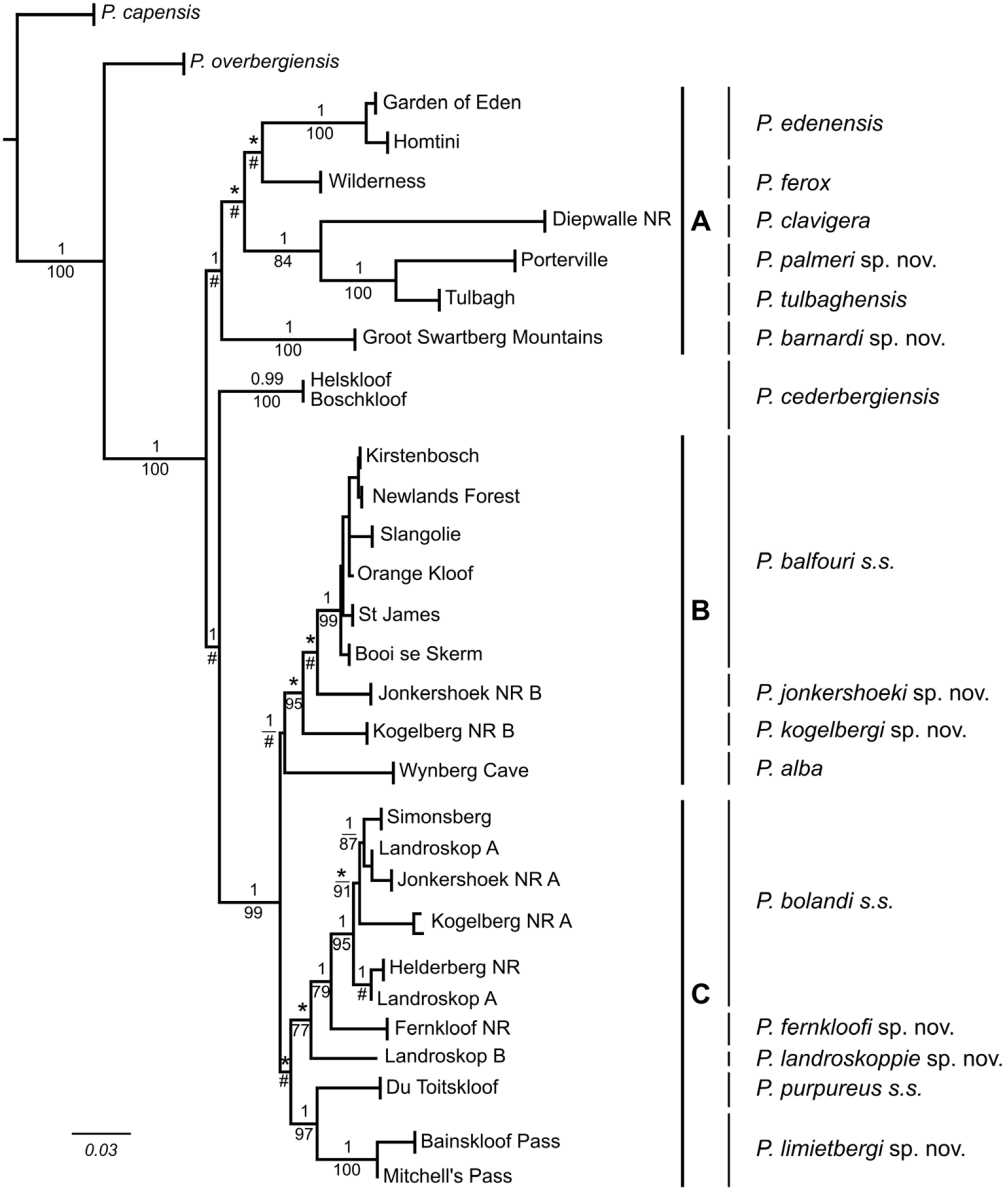
A maximum likelihood (ML) tree topology derived from the combined *COI* and *18S rRNA* data showing three clades (A–C). Bootstrap values (%) for the ML analyses is provided above the node. Only bootstrap values > 75% are shown. Below the node are the posterior probability (*p*) values derived from the Bayesian analyses. Only pP values > 0.95 are shown.

A 710 bp fragment of the *18S rRNA* locus was amplified for the Porterville (in the Groot Winterhoek Mountains) and the Groot Swartberg Mountains specimens. Sequences were deposited in GenBank (Accession numbers PP554397–PP554398). The specimens sequenced for the *18S rRNA* were combined with a single representative *COI* sequence for the same specimen and yielded a combined 1317 bp fragment. Phylogenetic analyses of the combined DNA sequence data using a ML and BI analyses produced the same three clades (A, B and C) evident from the *COI* analyses (Figure 2) hence, only the ML tree topology is shown and discussed (Figure 3). Clade A comprised *P. barnardi* **sp. nov**. from the Groot Swartberg Mountains in the Little Karoo, sister group to a southern Cape clade where *P. clavigera* was retrieved as sister group to *P. tulbaghensis* and *P. palmeri* **sp. nov**. from Porterville, Groot Winterhoek Mountains. *Peripatopsis cederbergiensis* was basal to clades B and C. Clade B was exclusively comprised of species from the western CFM, including the Cape Peninsula/Kogelberg Mountain clade species being sister group to Hottentots Holland/Limietberg Mountain species in clade C. In clade B, the Cape Peninsula *P. balfouri s.s*. was sister group to *P. jonkershoeki* **sp. nov**., and *P. kogelbergi* **sp. nov**., and the latter species was sister group to *P. alba* . In clade C, *P. purpureus s.s*. from Du Toitskloof was sister to *P. limietbergi* **sp. nov**.; the latter species formed a clade sister group to *P. landroskoppie* **sp. nov**., sister to *P. fernkloofi* **sp. nov**., sister group to *P. bolandi s.s*.

The uncorrected ‘*p*’ distance based on the *18S rRNA* revealed lower values compared to the *COI* locus due to the conservative nature of the locus. For example, between *P. balfouri s.s*. and *P. alba* the uncorrected distance was 0.61%; while between *P. balfouri s.s*. and *P. cederbergiensis* the uncorrected distance was 1.07%. Similarly, between *P. fernkloofi* **sp. nov**., and *P. landroskoppie* **sp. nov**., the uncorrected distance was 1.23%; while between *P. purpureus s.s*. and *P. limietbergi* **sp**. **nov**., the uncorrected distance was 1.51%. Finally, between *P. tulbaghensis* and *P. palmeri* **sp. nov**., the uncorrected distance was 2.54%.

### 3.3 Divergence Time Estimation

The most recent common ancestor (MRC) to the ingroup diverged 18.10 million years ago [Mya] [95% HPD 19.77–39.56 Mya], while *Peripatopsis tulbaghensis* diverged from its sister species *P. palmeri* **sp. nov**., 6.05 Mya [95% HPD 5.70–12.86 Mya]. *Peripatopsis barnardi* **sp. nov**., from the Groot Swartberg Mountains, diverged 15.02 Mya [95% HPD 13.62–28.09 Mya] from its MRC with the western CFM species. In the exclusively western CFM clade, *P. cederbergiensis* diverged 12.47 Mya [95% HPD 13.18–25.74 Mya] from the remaining Western Cape species, while *P. alba* from the Wynberg Caves on the Cape Peninsula/Table Mountain diverged 7.28 Mya [95% HPD 9.38–17.83 Mya] from *P. kogelbergi* **sp. nov**., clade. The latter species diverged 4.96 Mya [95% HPD 4.92–10.43 Mya] from *P. jonkershoeki* **sp. nov**., which diverged 3.65 Mya [3.33–7.24 Mya] from *P. balfouri s.s*. on the Cape Peninsula. Clades B and C diverged 8.81 Mya [95% HPD 8.72–16.33 Mya]. Within clade C, *P*. *landroskoppie* **sp. nov**., diverged from the remaining species 6.11 Mya [95% HPD 5.99–12.02 Mya] while *P. fernkloofi* **sp. nov**., diverged 4.60 Mya [95% HPD 4.25–8.99 Mya] from *P. bolandi s.s*. in the Hottentots Holland Mountain clade, while *P. purpureus s.s*. diverged from *P. limietbergi* **sp. nov**., 5.23 Mya [95% HPD 5.06–10.98 Mya].

### 3.4 Species Delimitation Using ASAP, bGYMC, bPTP and STACEY

**FIGURE 4:**
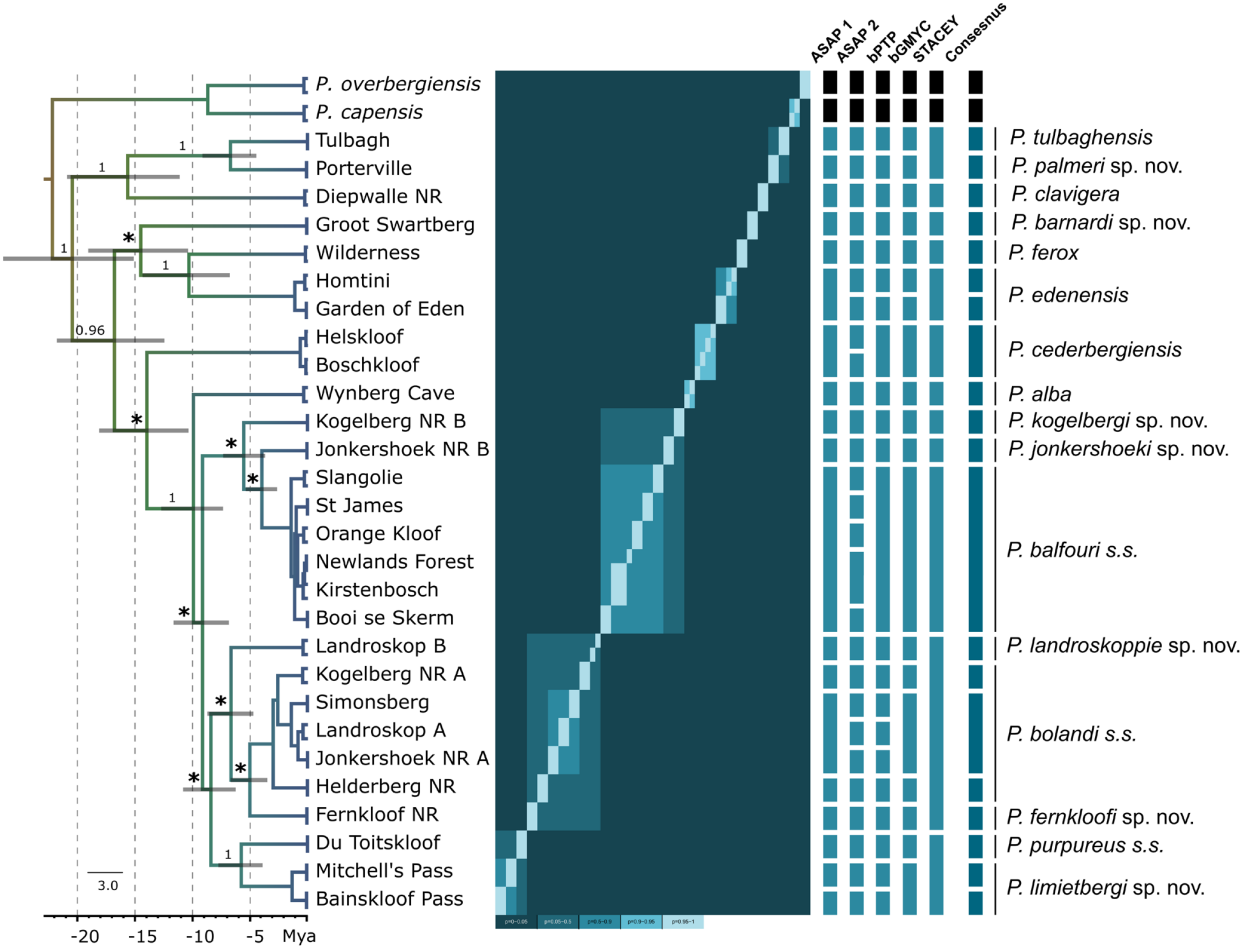
Beast chronogram derived from the combined *COI* and *18S rRNA* dataset demonstrating evolutionary relationships among the focal velvet worm taxa. Posterior probability values are shown above branches with non‐significant values (*p* < 0.95) indicated by an asterisk (*). Node bars show 95% highest posterior distributions for each divergence date estimate. The similarity matrix represents the results of the bGMYC analysis. The results of the four species delimitation methods (ASAP, bPTP, bGMYC, and STACEY) as well as the consensus between methods are indicated by the blue coloured bars to the right of the matrix.

The results of the four species delimitation methods were largely congruent, whereby variation can be explained by the different datasets and methodologies employed, which led to oversplitting and the recognition of intraspecific diversity (Figure 4). The two partitions retrieved from the automatic partitioning model (ASAP) analysis identified 22 and 36 distinct putative species from the *COI* dataset, respectively. Similarly, the Bayesian implementation of the Poison tree process (bPTP) identified 25 putative species from the *COI* dataset.

To delimit species based on the generalised mixed Yule‐coalescent (GMYC) output, a threshold needs to be established at which individuals could be considered conspecific. The results indicated by the similarity matrix in Figure 4 were interpreted at *p* < 0.9, which identified 19 putative species. A lower number was expected here based on the variation in the number of specimens included between the two datasets (see methods). By majority, the bGMYC analysis was congruent with the first ASAP partition and the bPTP results, albeit with less overestimation of species, which can be attributed to the inclusion of the conserved *18S rRNA* locus (Figure 4).

When defining localities as minimum clusters, the STACEY analysis retrieved 12 putative species (Figure 4). Upon visual inspection of the similarity matrix, the result of these 12 putative species identifying as independent clusters is well supported and was in the most part congruent with the former analyses. However, the STACEY analysis grouped geographically proximate and more closely related taxa more readily, which led to broader putative species identifications. Additionally, the phylogeny generated by the STACEY analysis (topology not shown) differed slightly from the chronogram (Figure 4) whereby Kogelberg NR B and Jonkershoek B were included in the same species cluster containing Landroskop B, Kogelberg Biosphere NR A, Simonsberg, Landroskop A, Jonkershoek NR A, Helderberg NR, and Fernkloof NR. This result is likely due to the varied ploidy levels for the two focal loci, which accounted for the varied substitution rates between them but likely placed a higher emphasis on the genetic structuring sourced from the conserved *18S rRNA* locus.

### 3.5 Morphology and Scanning Electron Microscopy (SEM)

**FIGURE 5:**
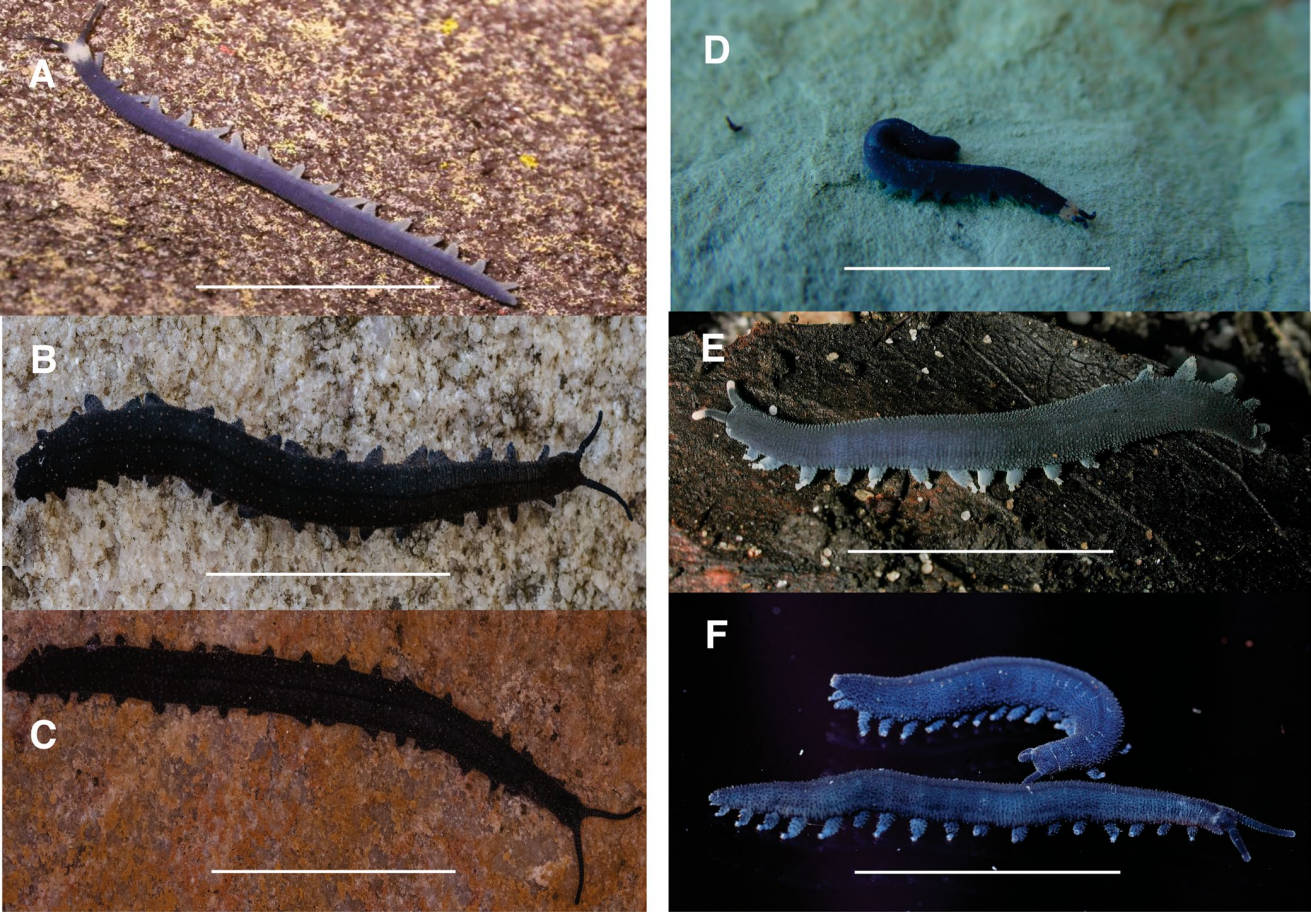
Photographic images of the dorsal aspect of selected live velvet worm species from the Western Cape province, South Africa (A–F*). Peripatopsis balfouri s.s*. from Bats Cave ravine, Cape Peninsula, Table Mountain range (A). *P. palmeri* **sp. nov**., from 22 Waterfalls, Porterville (B). *P. barnardi* **sp. nov**., from the Groot Swartberg Mountains, Little Karoo (C). *P. landroskoppie* **sp. nov**., from Landroskop (B) outside Grabouw (D). *P. fernkloofi* **sp. nov**., from Fernkloof Nature Reserve, Hermanus (E) and *P. limietbergi* **sp. nov**., from Mitchell's Pass (F). Scale bar = 10 mm.

Gross morphological characters such as leg pair numbers were of limited diagnostic value. For example, in both *P. palmeri* **sp. nov**., and *P. limietbergi* **sp. nov**., the number of leg pairs ranged from 16 to 18 (Table 2). In *P. purpureus s.s*., the number of leg pairs was 17, while in the sister species, *P. limietbergi* **sp. nov**., the number of leg pairs ranged from 16 to 18. In *P. landroskoppie* **sp. nov**., and the sympatric *P. bolandi s.s*. at Landroskop, the number of leg pairs was 18 (Table 2). It should be noted that the low sample sizes for several of the novel lineages (e.g., *P. landroskoppie* **sp. nov**., *P. fernkloofi* **sp. nov**., and *P. limietbergi* **sp. nov**.,) limits intraspecific comparison of leg pair numbers. Dorsal integument colour of live specimens was useful as a possible diagnostic tool (Figure 5A–F). For example, *P. fernkloofi* **sp. nov**., was dorsally royal blue and could be easily discerned from sympatric populations of *P. lawrencei* , which varied from rusty orange to slate black. Similarly, *P. landroskoppie* **sp**. **nov**., was dorsally royal blue, with a white head band, and could be discerned from the sympatric *P. bolandi s.s*., which is slate black in colour at Landroskop. While *P. purpureus s.s*. was slate black dorsally and could be discerned from *P. limietbergi* **sp**. **nov**., where specimens were blue dorsally.

**TABLE 2:** Velvet worm species (*Peripatopsis*), distribution, *N* (number of samples) with selected morphological characters.

Species	Distribution	*N*	Length (mm)	Width (mm)	Dorsal colour	Ventral colour	Leg pairs
*P. balfouri s.s*.	Cape Peninsula and adjacent interior	23	9.05–22.17	1.90–4.80	Black/purple	Pearl white	17–19
*P. purpureus s.s*.	Du Toitskloof	9	13.02–25.61	2.00–2.08	Slate black	Pearl white	17
*P. bolandi s.s*	Hottentots Holland, Simonsberg	18	18.22–24.09	3.60–5.20	Slate black	Pearl white	17–18
*P. barnardi* **sp. nov**.,	Swartberg Mountains, Little Karoo	9	23.88–16.60	3.30–2.24	Slate black	Light brown	17–18
*P. palmeri* **sp. nov**.,	Porterville	6	20.32–16.08	4.04–2.77	Slate black	Light brown	16–18
*P. limietbergi* **sp. nov**.,	Bainskloof Pass and Mitchel'ls Pass	5	21.03–14.04	4.36–2.04	Royal blue	Pearl white	16–18
*P. landroskoppie* **sp. nov**.,	Landroskop B	1	16.46	1.30	Royal blue	Pearl white	18
*P. fernkloofi* **sp. nov**.,	Fernkloof Nature Reserve	3	35.22–12.05	1.80–5.45	Royal blue	Pearl white	16
*P. jonkershoeki* **sp. nov**.,	Jonkershoek Nature Reserve B	4	14.57–12.63	2.84–2.78	Slate black	Pearl white	18
*P. kogelbergi* **sp. nov**.,	Kogelberg Biosphere Nature Reserve B	1	16.73	3.71	Slate black	Pearl white	18

**FIGURE 6:**
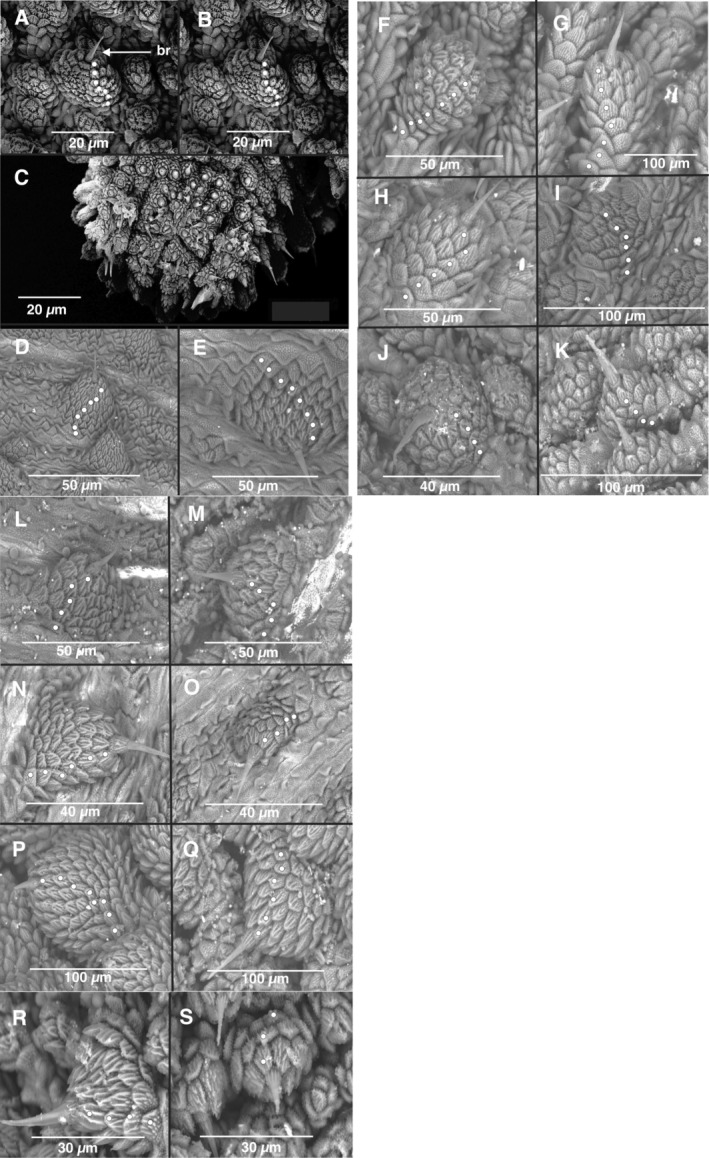
Scanning electron micrograph of the primary dermal papillae on the dorsal and ventral surfaces of the respective velvet worm species. A white dot represents a single scale rank. Dorsal papilla with sensory bristle (br) (A) of *Peripatopsis balfouri s.s*. and male genital opening surrounded by tumid lips (C). Dorsal papilla (B) for *P. bolandi s.s*. Dorsal papilla (D) and ventral papilla (E) for *P. purpureus s.s*., from Du Toitskloof. Dorsal papilla (F) and ventral papilla (G) for *P. palmeri* **sp. nov**. Dorsal papilla (H) and ventral papilla (I) for *P. barnardi* **sp. nov**. Dorsal papilla (J) and ventral papilla (K) for *P. landroskoppie* **sp. nov**. Dorsal papilla (L) and ventral papilla (M) for *P. fernkloofi* **sp. nov**. Dorsal papilla (N) and ventral papilla (O) for *P. limietbergi* **sp. nov**. Dorsal papilla (P) and ventral papilla (Q) for *P. jonkershoeki* **sp. nov**. and finally, the dorsal papilla (R) and ventral papilla (S) for *P. kogelbergi* **sp. nov**. Scale bars in micrometres (μm) are shown directly on micrographs.

**FIGURE 7:**
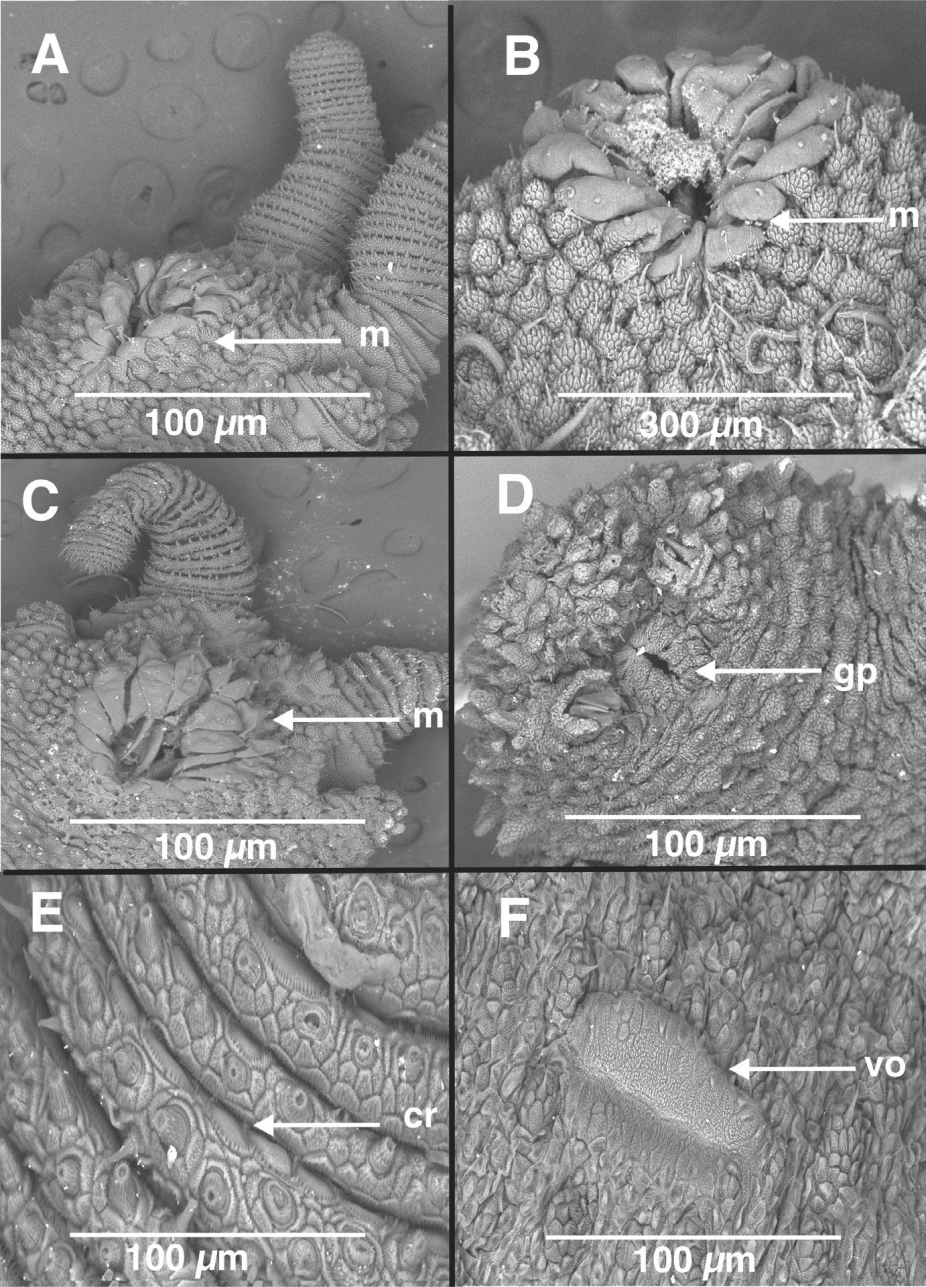
(A) Mouth (m) showing the arrangement of the lips in *Peripatopsis palmeri* **sp. nov**., *P. landroskoppie* sp. nov. (B), and *P. barnardi* **sp. nov**. (C). Male genital pore (gp) surrounded by tumid lips in *P. barnardi* **sp. nov** (D). The chemoreceptors (cr) on the antenna in (E) and finally the ventral organ (vo) in *P. limietbergi* **sp. nov** (F). Scale bars in micrometres (μm) are shown directly on micrographs.

Scanning electron microscopy (SEM) of the dorsal and ventral dermal papillae (Figure 6A–S) facilitated the delineation of the genetically recognized species (Figures 2, 3, 4). For example, the sister species *P. purpureus s.s*. and *P. limietbergi* **sp. nov**., can be differentiated by a fixed number of scale rank differences. Similarly, *P. bolandi s.s*. can be differentiated from *P. landroskoppie* **sp. nov**., in dorsal scale rank counts at Landroskop. While *P. tulbaghensis* and *P. palmeri* **sp. nov**., could also be differentiated by dorsal and ventral dermal papillae scale rank counts. Additional SEM of the oral papillae was not diagnostic (Figure 7A–C), while the genital opening of *P. barnardi* **sp. nov**., was observed (Figure 7D). The chemoreceptors of *P. limietbergi* **sp. nov**., were also observed between the antennal folds (Figure 7E). We report here, for the first time, the presence of the ventral organ (Figure 7F) in *P. limietbergi* **sp. nov**., suggesting it might be an autapomorphic character since we did not observe the character in any other velvet worm species during the present study.

## 4 Discussion

Refinement of species boundaries in the three species complexes (*Peripatopsis balfouri s.s*., *P. bolandi s.s*. and *P. purpureus s.s*.) coupled with additional sampling, phylogenetic analyses based on combined mt and nu DNA sequence data, SEM, and to a limited degree, with the dorsal integument colour of live specimens provides evidence for the recognition of seven novel velvet worm species. We used the species delimitation methods as a guide in the recognition of novel diversity. Both iNaturalist records from the two previously unsampled localities were corroborated to be new species. Five of the novel recognized species are narrow range endemics, such as, for example, *P. barnardi* **sp. nov**., and *P. palmeri* **sp. nov**., while several species could not be recollected (*P. fernkloofi* **sp. nov**., and *P. landroskoppie* **sp. nov**.,) despite exhaustive attempts, presumably due to their rarity. Although additional sampling should be conducted to investigate the distribution of these new species. In contrast, during the present study, at least two species were confined to large CFM blocks, such as, for example, *P. balfouri s.s*., and *P. bolandi s.s*., which are present on the Cape Peninsula and Hottentots Holland Mountains, respectively. Thus, the results of the present study, coupled with previous systematic studies of *Peripatopsis* (McDonald et al. 2012; Daniels et al. 2013; Ruhberg and Daniels 2013; Barnes et al. 2020; Grobler et al. 2023; Barnes and Daniels 2024; Nieto Lawrence and Daniels 2024) negate the observation by Oliveira (2023) that species names be restricted to type localities; clearly, this is a gross oversimplification. Furthermore, the premise that a single authority exists for Onychophora ignores local researcher expertise who actively work on their country's fauna and reflects a colonial mindset that is futile. The complexities of species delimitation in velvet worms are furthermore clearly illuminated by a recent study on the *P. sedgwicki* species complex, where the combined use of RADseq and DNA sequence (*COI* and *18 S rRNA*) data plus SEM data and limited morphological characters supported the presence of three novel species (Myburgh et al. 2024; Barnes and Daniels 2024). The latter studies demonstrated high levels of congruence between mt and nu DNA tree topologies in the *P. sedgwicki* species complex, suggesting that while *18S rRNA* is conserved, it is variable enough to corroborate mt DNA differentiation. During the present study, the *18S rRNA* locus was also found to be informative. Traditional alpha‐taxonomic characters, such as leg pair numbers, were not useful to differentiate the novel species from their sister species (Figure 3; Table 2). However, the dorsal integument colour of live specimens was a useful infield taxonomic character with which to delineate most of the novel species, particularly where species occur in sympatry with congenerics (Figure 5A–F). What the evolutionary drivers are of the bright blue on sister species at high (*P. landroskoppie* **sp. nov**., and *P. limietbergi* **sp. nov**.,) and low altitudes (*P. fernkloofi* **sp. nov**.,) remain unknown. Scanning electron microscopy revealed fixed differences in dorsal and ventral scale rank counts of the dermal papillae among sister species pairs.

Our divergence time estimation revealed the Miocene epochs as a major driver for cladogenesis among ingroup velvet worm lineages. During the early Miocene, climatic conditions remained relatively stable in South Africa up to the end of the middle Miocene climatic optimum (MMCO) which occurred roughly 20–15 Mya, albeit with intermittent amelioration (Zachos et al. 2001; Cowling et al. 2009; Frigola et al. 2018). Mesic conditions under the MMCO likely facilitated the colonisation of large CFM mountain blocks with the initial diversification of ancestral lineages. Post the MMCO, the climate conditions deteriorated during the middle Miocene climatic transition (MMCT), which resulted in decreasing global temperatures and ice sheet expansions, resulting in forest fragmentation, further promoting cladogenesis in the velvet worm ancestral species. During the ensuing aridification of the MMCT, coupled with the formation of the proto Benguela current and two geotectonic uplift events that altered the edaphic landscape, led to the contraction of the Afrotemperate forest biome and its restriction to deep gorges along the CFM belt (Tyson and Partidge 2000; Dupont et al. 2011).

Marine transgressions flooded coastal regions, in some areas with up to 150 m which would effectively isolate mountain blocks and result in the extinction of low‐lying invertebrate communities and their forested habitat. Under these conditions, the ancestral velvet worm species would have become restricted to high altitude regions along the CFM in the Western Cape. Aridification continued and intensified throughout the Mio/Pliocene, during which time forests became confined to refugial areas in deep gorges at high altitude along the CFM (Forest et al. 2007; Daniels et al. 2013, 2017). McDonald and Daniels (2012) demonstrated the Plio/Pleistocene climatic variation induced divergence between *P. capensis* , *P. overbergiensis* McDonald et al. 2012 and *P. lawrencei* , resulting in the species being confined to three allopatric clades. Myburgh and Daniels (2022) explored cladogenetic drivers among ectothermic invertebrates in the Western Cape and concluded that climate/temperature and its impact on habitat availability were the most crucial abiotic factors, together with mountain barriers that promote speciation in the region. Daniels et al. (2024), while examining the drivers of speciation in an endemic CFM freshwater crab clade (*Potamonautes* MacLeay 1838), revealed that temperature and rainfall regimes were the main abiotic factors promoting speciation.

The question now arises as to what evidence exists for the recognition of new species. During the present study, the mitochondrial *COI* sequence distance ‘*p*’ values between sister species pairs exceeded > 6%. This value is within the range reported for other *Peripatopsis* sister species pair groups (McDonald and Daniels 2012; Grobler et al. 2023; Nieto Lawrence and Daniels 2024). For example, interspecific *COI* sequence distance values between *P. birgeri* and *P. polychroma* was 7.40% (Grobler et al. 2023). Similarly, between *P. lawrencei* and *P. overbergiensis* the distance values was 8.39%, while between *P. capensis* and *P. overbergiensis* a distance of 6.58% was reported (McDonald and Daniels 2012; Nieto Lawrence and Daniels 2024). Intraspecific sequence values between three allopatric populations of *P. overbergiensis* was 2.97% (Myburgh and Daniels 2015), while in *P. lawrencei* intraspecific distances was 3.91% (McDonald and Daniels 2012; Nieto Lawrence and Daniels 2024); while between allopatric populations of *P. birgeri* and *P. The polychroma* distance values were 2.73% and 5.86%, respectively (Grobler et al. 2023). We interpret the marked uncorrected interspecific *COI* distance values of > 6% reported in the present study as corroborative evidence for genetic isolation, particularly among sympatric species. Furthermore, the uncorrected sequence distances derived from the *18S rRNA* were high, ranging from 0.61% to 2.54%, providing nuclear DNA evidence for the recognition of the novel lineages. Sato et al. (2018) reported very low levels of genetic differentiation in the Australian velvet worm genus *Kumbadjena* (Reid 2002) while describing novel species. Similarly, Barnes et al. (2020) reported uncorrected sequence distance values of 0.68% in the *P. clavigera* species complex for the *18S rRNA* locus. The marked nuclear and mitochondrial sequenced distances observed in the present provides evidence for reproductive isolation.

Species delimitation methods revealed a moderate degree of congruence between the various analytical approaches, although with variation in the number of putative species identified (Figure 4). However, all four species delimitation methods recognised *P. barnardi* **sp. nov**., *P. kogelbergi* **sp. nov**., *P. jonkershoeki* **sp. nov**., and *P. palmeri* **sp. nov**., as distinct putative species. In the aforementioned instances, there is complete congruence with our recognition of these species as distinct lineages, a result confirmed by fixed gross morphological (dorsal integument colour) scale rank count differences and geographic exclusivity in conjunction with the combined DNA sequence tree topology (Figure 3). In several instances, the species delimitation methods overestimated the number of species, a pattern that is widespread in taxa characterised by limited dispersal capabilities, where pronounced genetic differentiation caused by genetic drift and fixation may artificially promote the recognition of additional species (Myburgh and Daniels 2015). We advocate for an integrative systematic approach when delineating species boundaries and suggest the incorporation of additional diagnostic characters derived from independent sources to validate species boundaries. In this regard, we argue against the callous interpretation of species delimitation methods' results. For example, we disagree with the designation of the two localities (Mitchell's Pass and Bainskloof Pass) where *P. limietbergi* **sp. nov**., occurs as two distinct species due to the low *COI* distance values (2.98%) and invariant dorsal or ventral scale papilla counts between these two localities. Additionally, the dorsal integument and leg pair counts were also invariant, corroborating our designation of a single species. Lord et al. (2024) using *COI* sequence data, recently employed these species delimitation methods to the two New Zealand velvet worm genera (*Peripatoides* Pocock 1894 and *Ooperipatellus* Ruhberg 1985) and demonstrated widespread cryptic diversity, corroborating our results. Notably, the latter study did not include nuclear markers, suggesting that their gene trees may potentially have overestimated the number of species.

Traditional alpha taxonomic characters such as leg pair number were of limited utility to differentiate the novel lineages. For example, the number of leg pairs for most groups ranged from 16 to 18. However, the dorsal integument colour of live specimens was very useful at delineating species in field, particularly where species occur in sympatry. Trewick et al. (2024) reported that in New Zealand, *Peripatoides indigo* (Ruhberg 1985) always had a distinct blue colour that differentiated it from other species. In *P. birgeri* from KwaZulu‐Natal, a white band behind the head was consistently present and aided the differentiation of the latter species from its sister species, *P. polychroma* (Grobler et al. 2023). Similarly, within *P. sedgwicki s.s*., fine‐scale studies revealed the presence of one clade that also possessed a white head band that was diagnostic for the clade (Barnes and Daniels 2024). In the Peripatidae Audouin and Milne‐Edwards (1832), a white head band is diagnostic for at least two velvet worm species: *Peripatus heloisae* (Carvalho 1941) and *Macroperipatus torquatus* (von Kennel 1883) suggesting that this character is potentially useful taxonomically when delineating species (Costa and Giribet 2021). Daniels and Ruhberg (2010) observed the presence of two distinct sympatric colour lineages of *P. moseleyi s*.*s*. in the Amathola Mountains, which were later described as two species (Ruhberg and Daniels 2013). However, dorsal integument colour is not always indicative of distinct species status. For example, in *P. overbergiensis*, distinct colour morphs are present at Grootvadersbosch Nature Reserve, which are genetically invariant (Myburgh and Daniels 2015). Similarly, in *P. lawrencei* , colour is highly polymorphic, ranging from bright orange to rusty brown to slate black, and no taxonomic differences have been observed between these colour morphs (Nieto Lawrence and Daniels 2024). The taxonomic value of colour as an initial diagnostic feature for species differentiation should, however, not be underestimated, and we recommend that live species be photographed prior to ethanol preservation.

Scanning electron micrograph results proved useful to delineate species boundaries, by demonstrating fixed dorsal and ventral scale‐count differences between sister *Peripatopsis* species groups during the present study (Figure 6A–S). We argue for an integrative use of colour as a possible tool for species diagnosis and its use in conjunction with other morphological characters, including DNA sequence data. Similarly, ecological differences should be noted in situ during collection as these may aid the recognition of lineages. Considering the marked DNA sequence and fixed diagnosis morphological characters we continently recognised seven novel species. We invoke the phylogenetic species concept, defined as “an irreducible group whose members are descended from the same ancestor” (de Queiroz 2007). We observed considerable fixed genetic and morphological differences between sister species groups, validating their evolutionary distinction and their recognition as species. To this end, we recognise seven novel velvet worm species that are described in the present study.

## 5 Taxonomy

**Phylum Onychophora Grube, 1853**.

**Family Peripatopsidae Bouvier, 1905**.

**Genus *Peripatopsis* Pocock** (1894).

***Peripatopsis balfouri s.s*. Sedgwick** (1885).

(Figures 2, 3, 5A, 6A,C; Table 2).

Neotype: 1 m (male), Booi se Skerm, 34°18′16′′S, 18°27′36′′E, Cape Point, Cape Peninsula, Western Cape province, South Africa, collected 8 December 2010 by D.E. McDonald and A. Abels (SAM‐ENW‐C006508).

Additional material: 3 f (female), Booi se Skerm, 34°18′16′′S, 18°27′36′′E, Cape Point, Cape Peninsula, Western Cape province, South Africa, collected 8 December 2010 by D.E. McDonald and A. Abels, (SAM‐ENW‐C006508); 2 f and 2 specimens sex not determined, Newlands ravine, 33°58′12′′S, 18°27′00′′E, Cape Peninsula, Western Cape province, South Africa, collected 9 December 2010 by D.E. McDonald and A. Abels, (SAM‐ENW‐C006553); 1 f and one specimen sex not determined, Newlands forest, 33°58′12′′S, 18°27′00′′E, Cape Peninsula, Western Cape province, South Africa, collected 2006 by S. R. Daniels, (SAM‐ENW‐C006554); 4 specimens sex not determined (same locality as before), collected 15 April 2006 by M. Picker and R. Cowlin (SAM‐ENW‐C006568); 1 f and 2 specimens sex not determined, Slangolie Ravine, 33°58′39′′S, 18°23′00′′E, Cape Peninsula, Western Cape province, South Africa, collected 7 September 2010 by D.E. McDonald and A. Abels, (SAM‐ENW‐C006513); 3 specimens sex not determined, St James, Kalk bay, Cape Peninsula, Western Cape province, South Africa 34°06′56′′S, 18°27′30′′ E, collected date unknown by D.E. McDonald and A. Abels, (SAM‐ENW‐C006514); 3 specimens sex not determined, Booi se Skerm, Cape Peninsula, Western Cape province, South Africa, collected 1995 by Robertson, (SAM‐ENW‐X1074); 2 specimens sex not determined, Simonstown, Cape Peninsula, Western Cape, South Africa, collected 1898 by W.F. Purcell (SAM‐ENW‐X4011); 1 specimen sex not determined, Newlands, Cape Peninsula, Western Cape province, South Africa, collected 1892 by Purcell, (SAM‐ ENW‐X1078); 3 specimens, sex not determined, Hout Bay, Cape Peninsula, Western Cape province, South Africa, collected 1898 by W.F. Purcell (SAM‐ENW‐X1080); 1 specimen sex not determined, Rondebosch, Cape Peninsula, Western Cape province, South Africa, collected 1898 by Treleaven (SAM‐ENW‐X1082); 2 specimens sex not determined, Platteklip Ravine, Cape Peninsula, Western Cape province, South Africa, collected 1896 by Purcell, (SAM‐ENW‐X1084); 4 specimens sex not determined, Devil's Peak, Cape Peninsula, Western Cape province, South Africa, collected 1900 by W.F. Purcell (SAM‐ENW‐X7273); 3 specimens sex not determined, St. James, Cape Peninsula, Western Cape province, South Africa, collected 1901 by W.F. Purcell (SAM‐ENW‐X1074); Simonstown, Cape Peninsula, Western Cape province, South Africa, collected 1898 by Purcell (SAM‐ENW‐X4011); 1 specimen, sex not determined, St James, Cape Peninsula, Western Cape, South Africa, collected 1901 by Purcell (SAM‐ENW‐C3654).

GenBank: *COI*: Kirstenbosch, EU 855343; Newlands, EU 855269, 855,271, 855,272, KC 766104– KC 766107 (Daniels et al. 2009); Slangolie, KC 766111–KC 766113; Booi se Skerm, KC 766116–KC 766120; St James, KC 766121–KC 766123; Orange Kloof, KC 766129–KC 766131 (Daniels et al. 2013): *18S rRNA*: Kirstenbosch, EU 855539 (Daniels et al. 2009), KC 766069; Simonsberg, KC 766070; Booi se Skerm, KC 766074; Newlands, KC 766075; Slangolie, KC 766076; St James, KC 766077; Orange Kloof, KC 766080 (Daniels et al. 2013).

Description. Neotype m: Length: 15 mm, Paratypes (f adults, *n* = 3): 9–22 mm (Daniels et al. 2013).

Leg pair number: 18 leg pairs, only the Newlands locality had a leg pair count ranging from 17 to 19 (Daniels et al. 2013).

Convex‐conical dermal papillae with six to nine dorsal scale ranks. Male crural papillae are semicircular (Figure 6A; Daniels et al. 2013).

Colour and patterning. Indigo‐blue with white papillae or rust brown‐black with white head collar (Figure 5A). Dorsal midline with two light‐coloured lateral bands above the legs and along the entire body. Bright white ventral surface.

Legs. 18 leg pairs with claws. The last leg pair is reduced and often highly reduced in males. Three complete spinous footpads. Male crural papillae are semicircular. Integument. Convex‐conical dermal papillae with 6–9 scale ranks (Figure 6A). Mouth surrounded by 11 oral lips. Male genital area. Gonopore star‐shaped with six genital pads. Genital pads without sensory spines (Figure 6C).

Habitat: Afromontane forest patches on mountains. Collected in river moss and decaying logs.

Distribution: Endemic to the Cape Peninsula, Table Mountain, and Simonsberg in the Western Cape province, South Africa. The species occurs at very low frequency outside of the Cape Peninsula.

***Peripatopsis bolandi s.s.* Daniels et al.** (2013).

(Figures 2, 3, 4, 6B; Table 2).

Holotype: 1 f, Simonsberg Mountains, 33°53′31′′S, 18°55′25′′E, Western Cape province, South Africa, collected 2 March 2011 by D.E. McDonald and A. Abels (SAM‐ENW‐C006510).

Paratype: 2 f, Simonsberg Mountains, 33°53′31′′ S, 18°55′25′′ E, Western Cape province, South Africa collected 2 March 2011 by D.E. McDonald and A. Abels (SAM‐ENW‐C006510).

Additional Material: 1 f, Simonsberg Mountains, 33°53′31′′S, 18°55′25′′E, Western Cape province, South Africa collected 2 March 2011 by D.E. McDonald and A. Abels (SAM‐ENW‐C006510); 1 f, Helderberg Nature Reserve 34°02′32′′S, 18°52′26′′E, Hottentots Holland Mountains, Western Cape province, South Africa, collected 29 September 2010 by D.E. McDonald and A. Abels (SAM‐ENW‐C006515); 2 f, Helderberg Nature Reserve, 34°02′32′′S, 18°52′26′′E, Hottentots Holland Mountains, Western Cape province, South Africa, collected 29 September 2010 by D.E. McDonald and A. Abels (SAM‐ENW‐C006576); 4 specimens sex not determined, Landroskop, 33°56′393′′S, 18°51′344′′E, Grabouw, Western Cape province, South Africa, collected 16 March 2006 by S.R. Daniels (SAM‐ENW‐C006562); 9 specimens (not sexed), Helderberg Nature Reserve, Hottentots Holland Mountains, Western Cape province, South Africa, same coordinates as earlier 29 September 2022, collected S.R. Daniels, (SAM‐ENW‐C015303); 4 specimens (not sexed), Kogelberg Biosphere Nature Reserve, 34°19′59′′S, 18°57′01′′E, Western Cape province, South Africa, collected 11 April 2021 by A. Myburgh and S.R. Daniels (SAM‐ENW‐C015302).

Description: Male, length 21 mm; females 18–22 mm (Daniels et al. 2013).

Colour pattern: Dorsally slate black to indigo, pearl white ventrally.

Leg pair number: 18 leg pairs (Table 2, Daniels et al. 2013).

Integument: convex‐conical dermal papillae with six to ten scale ranks dorsally (Figure 6B, Daniels et al. 2013).

Genital opening: Gonopore star‐shaped.

GenBank: *COI*, Landroskop, EU 855306–EU 8553010; Kogelberg Biosphere Nature Reserve, EU 855337–EU 855338, Helderberg Nature Reserve, EU 855301‐EU 855305, KC 766108–KC 766110; Jonkershoek Nature Reserve site 2, EU 855324–EU 855326; EU 855328; Simonsberg Mountains, KC 766096–KC 766099, KC 766101–KC766103 (Daniels et al. 2009), Helderberg Nature Reserve, PP 550038–PP 550044 (present study), Kogelberg Biosphere Nature Reserve, PP 550035–PP 550037 (present study); *18S rRNA*, Landroskop EU 855537, KC 766084, Kogelberg Biosphere Nature Reserve, EU 855538 (Daniels et al. 2009), KC 766087; Simonsberg Mountains KC 766073 (Daniels et al. 2013).

Etymology: Named after the Boland region of the Western Cape province, South Africa.

Distribution: Present in small Afrotemperate forest patches along the Hottentots Holland, Landroskop (A), Jonkershoek Nature Reserve (A) and Kogelberg Biosphere Nature Reserve (A) where it occurs in close proximity to streams.

Habitat: The species is present in decaying indigenous logs of wood, dead reed beds (*Restio*) and leaf litter in high altitude Afrotemperate forest patches.

Remarks: At Jonkershoek Nature Reserve, *P. lawrencei* is present at lower altitudes on the Jonkershoek valley floor (McDonald et al. 2012), while *P. jonkershoeki* **sp. nov**., is present at higher altitudes. *Peripatopsis bolandi s.s*. is sympatric with *P. kogelbergi* **sp**. nov. at Kogelberg Biosphere Nature Reserve.

***Peripatopsis purpureus s.s.* Daniels et al.** (2013).

(Figures 1, 2, 3 and 6D–E; Table 2).

Holotype: 1 m, Du Toitskloof (Mollenaars Hike), 33°42′43′′S, 19°06′33′′E, Western Cape province, South Africa, collected 20 January 2011 by D.E. McDonald and A. Abels (SAM‐ENW‐C006509‐ one tube with one specimen).

Paratype: 2 f, Du Toitskloof (Mollenaars hike), 33°42′43′′S, 19°06′33′′E, Western Cape province, South Africa, collected 20 January 2011 by D.E. McDonald and A. Abels (SAM‐ ENW‐C006509‐one tube with two specimens).

Additional Material: 4 m and 1 f, Du Toitskloof (Mollenaars hike), 33°42′ 43′′S, 19°06′ 33′′E, Western Cape province, South Africa collected 20 January 2011 by D.E. McDonald and A. Abels, (SAM‐ENW‐C006509‐ one tube 5 specimens); 4 specimens (not sexed), Du Toitskloof (Mollenaars hike), Western Cape province, South Africa, same as above, collected 24 May 2021 by A. Myburgh and S.R. Daniels (SAM‐ENW‐C015305).

Description: Holotype measurements 17 mm; females 22–35 mm (Daniels et al. 2013).

Colour pattern: Dorsally slate black and pearl white ventrally (Daniels pers. obs.).

Leg pair number: 17 leg pairs, last leg pair highly reduced (Table 2, Daniels et al. 2013).

Integument: Dermal papillae concave‐conical with sensory bristle. Seven dorsal and eight ventral scale ranks (Figure 6D,E).

Genital opening: Gonopore star‐shaped.

GenBank: *COI*, Du Toitskloof, KC 766124–KC 766127 (Daniels et al. 2013), PP 550031–PP 550033 (present study); *18S rRNA*, Du Toitskloof, KC 766078 (Daniels et al. 2013).

Etymology: Named after the purple‐blue colour of the integument while alive, when it included the specimens from Bainskloof Pass and Mitchells' Pass. The specimens from the latter two localities are transferred to *P. limietbergi* **sp**. **nov**., that are herein described. The specimens from Du Toitskloof (Mollenaars hike) are black dorsally and white ventrally. To limit future confusion, we restricted the species name exclusively to the type locality, Du Toitskloof.

Distribution: Only present along the riverbank in a deep valleys or ravines where the water cascades, keeping the moss banks moist, at the end of the Mollenaars hike, Du Toitskloof, Western Cape province, South Africa.

Habitat: Collected under vegetation and moss carpets along the banks of the stream as well as in soil under rocks.

Remarks: Marked uncorrected sequence ‘*p*’ distances evident for both the mitochondrial *COI* and nuclear *18S rRNA* loci exist between *P. purpureus s.s*. and its sister species *P. limietbergi* **sp. nov**. In addition, *P. limietbergi* **sp. nov**., has a distinct number of ventral and dorsal scale ranks, further distinguishing it from is sister species, *P. purpureus s.s*. In the study by Daniels et al. (2013) there is strange clustering of one Du Toitskloof animal in the *P. balfouri s.s*. clade, this is likely a sample that was swapped during sequencing. *Peripatopsis purpureus s.s*. is now restricted exclusively to the Du Toitskloof sample locality.

***Peripatopsis palmeri* sp. nov**.,

urn:lsid:zoobank.org:act:651E8CAB‐6A13‐4163‐A915‐F0C005453385.

(Figures 1, 2, 3, 4, 5B, 6F–G, 7A; Table 2).

Holotype: 1 f, 22 Waterfalls, Porterville, 32°59′0.007″S, 19°01′0.767″ E, Western Cape Province, South Africa; collected 26 February 2022by S.R. Daniels, A. Barnes, F. Gordon, and P.C. Grobler, 274 m above sea level (ASL) (SAM‐ENW‐C015313).

Paratype: 1 m, same collection information as the holotype (SAM‐ENW‐C015314).

Additional Material: 2 adult males, same collection information as for holotype (SAM‐ENW‐C015315); 2 juveniles (not sexed) collected at the campsite at 22 Waterfalls, same collection information as the holotype (SAM‐ENW‐C015312).

Description: Holotype measurements: 20.32 mm length, 4.05 mm width; males ranged from 18.24 mm length, 2.66 mm in width to 16.51 mm length, 2.87 mm in width; female ranged from 16.06 mm in length to 2.77 mm in width (Table 2).

Colour pattern: Dorsally slate black, with a prominent black mid dorsal line (Figure 5B). Ventrally off white with light brown.

Leg pair number: 17 leg pairs in holotype with a claw on the last reduced leg pair; leg pairs varied from 16 to 18, last leg pair reduced, sometimes without a claw (Table 2). Mouth with lips (Figure 7A).

Integument: Primary dermal papillae with seven dorsal and seven ventral scale ranks (Figure 6F–G).

Genital opening: Gonopore star‐shaped.

GenBank: *COI*, PP 550049–PP 550054 (present study); *18S rRNA*, PP 554398 (present study).

Etymology: Named after Caleb Palmer and his mother, Nici Palmer, who first observed the species and posted the record to iNaturalist. Named for their shared surname.

Distribution: Narrow endemic, along stream bank at Porterville, Groot Winterhoek Mountains, Western Cape province, South Africa.

Habitat: Under moss and stones adjacent to the stream bed at 22 Waterfalls, Porterville, Western Cape province, South Africa. The area is characterized by indigenous fynbos and trees such as the wild olive ( *Olea europaea* subs *africana*) that occur along the stream banks.

Remarks: *Peripatopsis palmeri* **sp. nov**., is sister group to *P. tulbaghensis* with marked uncorrected distances of 9.22% and 2.45% for the *COI* and *18S rRNA* loci respectively, despite the close geographic proximity of these two species. *Peripatopsis palmeri* **sp. nov**., is further distinct from *P. tulbaghensis* based on dorsal scale counts of seven and five respectively (Barnes et al. 2020). The white epigeal form of the species first documented on iNaturalist most likely represents a juvenile.

***Peripatopsis barnardi* sp. nov**.,

urn:lsid:zoobank.org:act:79D0B854‐CDC0‐4E01‐B4F7‐41AEE88657EF.

(Figures 1, 2, 3, 4, 5C, 6H–I, 7C–D; Table 2).

Holotype: 1 m, farm of G. A. Steenkamp Groot Swartberg Mountains between Calitzdorp and Oudtshoorn, Little Karoo, 33°22′614″ S, 22°02′176″ E, Western Cape province, South Africa, collected 6 March 2022 by R. Barnard, 813 m ASL (SAM‐ENW‐C015319).

Paratype: 2 m, same locality as above, collected 2 July 2022 by S.R. Daniels, A. Barnes, A. J. Nieto Lawrence and P.C. Grobler (SAM‐ENW‐C015320).

Additional Material: 9 specimens (7 adults and 2 juveniles, not sexed) same locality and collection information as for the paratype (SAM‐ENW‐C015318).

Description: Holotype measurements, 19.19 mm in length, 3.27 mm in width (Table 2).

Additional material: 23.88 mm in length and 3.30 mm in width.

Colour pattern: Dorsally slate black, with a prominent black mid dorsal line (Figure 5C), ventrally light brown. Mouth with lips (Figure 7E).

Leg pair number: 17 to 18 leg pairs, with a claw on the last leg pair (Table 2).

Integument: Primary dermal papillae with six dorsal and six ventral scale ranks (Figure 6H–I).

Genital opening: Gonopore (Figure 7D).

GenBank: *COI*, PP 550045– PP550048 (present study); *18S rRNA*, PP 554397 (present study).

Etymology: Named after Mr. Rohan Barnard, who collected the first specimen of the new species while looking for ants and posted the record to iNaturalist.

Distribution: Narrow endemic, likely present in ancient isolated Afrotemperate forest patches along the southern flanks of the Groot Swartberg Mountains in the Little Karoo, Western Cape province, South Africa.

Habitat: Along the periphery of the Hotnots river, under smooth river rocks/boulders covered in deep leaf litter where the species was found in the soil. Historically, the area was likely an Afrotemperate forest patch, evident from the remnant indigenous tree growth along and in the stream. However, the riverbanks have been transformed by alien (*Acacia*) vegetation (Daniels per. obs.).

Remark: Phylogenetically, basal to species from the southern Cape species ( *P. clavigera* , *P. ferox* and *P. edenensis*) and the Groot Winterhoek Mountain species ( *P. tulbaghensis* and *P. palmeri* **sp. nov**.,) Several deep gorges are present along the Groot Swartberg Mountains that likely harbour additional populations of *P. barnardi* **sp. nov**. More sampling is required to document the distribution of the species, such as for example, the forested upper reaches of the stream at the Swartberg Backpackers, the Matjesriver, and Dassieklipriver. This is the first species from the Klein Karoo, a very arid region in the interior of the Western Cape province.

***Peripatopsis landroskoppie* sp. nov**.,

urn:lsid:zoobank.org:act:D06D24C9‐1A83‐4C0F‐8E90‐798BB20751CC.

(Figures 1, 2, 3, 4, 5D, 6J,K, 7B; Table 2).

Holotype: 1 specimen (not sexed), Landroskop, 33°56′393′′S, 18°51′344′′E, Grabouw, Western Cape province, collected 16 March 2006 by S.R. Daniels (SAM‐ENW‐C015321).

Description: Holotype measurements 13.45 mm length, 2.25 mm width (Table 2).

Colour pattern: Dorsally blue, with a white head (Figure 5 D), ventral pearl white.

Leg pair number: 17 leg pairs, last pair with claw (Table 2). Mouth with lips (Figure 7B).

Integument: Primary dermal papillae, four dorsal and four ventral scale ranks (Figure 6J–K).

Genital opening: Gonopore star‐shaped.

GenBank: *COI*, EU 855349 (Daniels et al. 2009).

Etymology: Named after Landroskop, a peak behind Grabouw.

Distribution: At present only known from the type locality, Landroskop (B), Western Cape province, South Africa.

Habitat: Collected from under decaying reed beds (Restionaceae) under a small overhanging rock ledge. Found in near sympatry with *P. bolandi s.s*. and *P. lawrencei* at Landroskop (Daniels pers. obs.).

Remarks: *Peripatopsis landroskoppi* **sp. nov**., is sympatric with *P. bolandi s.s*. at Landroskop (Daniels et al. 2013). *Peripatopsis lawrencei* is also present at Landroskop (Daniels pers. obs.). The white head and bright blue dorsal colour of *P. landroskoppi* **sp. nov**., easily differentiate it from both *P. bolandi s.s*. and *P. lawrencei* , which are both slate black at Landroskop (Daniels pers. obs.). *Peripatopsis landroskoppi* **sp. nov**., appears remarkably rare since it could not be recollected despite exhaustive search attempts and is of conservation concern. Additional sampling is required in the future to determine the species distribution.

***Peripatopsis fernkloofi* sp. nov**.,

urn:lsid:zoobank.org:act:61305BF2‐7844‐4C8F‐A774‐95BB22F8587A.

(Figures 1, 2, 3, 4, 5E, 6L,M; Table 2).

Holotype: 1 m, Fernkloof Nature Reserve, 34°23′37′′S, 19°16′34′′E, Hermanus, Western Cape province, South Africa, collected 11 May 2006 by S.R. Daniels and H. van den Worm (SAM‐ENW‐C015309).

Paratype: 1 juvenile specimen (not sexed), same locality and collectors information as holotype (SAM‐ENW‐C0015308).

Additional material: 1 f, same locality and collectors information as for holotype (SAM‐ENW‐C006555).

Description: Holotype measurements, 35.22 mm length, 5.45 mm width.

Colour pattern: Dorsally royal blue, ventrally white when alive (Figure 5E), with white‐tipped antennae.

Leg pair number: 16 in holotype, with a claw on the last leg pair.

Integument: Primary dermal papillae with five dorsal and five ventral scale ranks (Figure 6L–M).

Genital opening: Gonopore star‐shaped.

GenBank: *COI*, Fernkloof Nature Reserve, EU 855333–EU 855335 (Daniels et al. 2009); *18S rRNA*, KC766086 (Daniels et al. 2013).

Etymology: Named after the type locality, Fernkloof Nature Reserve where the species is found.

Distribution: Narrow endemic, confined to Fernkloof Nature Reserve, Hermanus, Western Cape province, South Africa.

Habitat: Under sandstone in thick leaf litter in a small Afrotemperate forest patch at Fernkloof Nature Reserve, Hermanus, below the municipal dam wall.

Remarks: *Peripatopsis fernkloofi* **sp. nov**., is found in near sympatry with *P. lawrencei* at Fernkloof Nature Reserve (Daniels et al. 2009; Nieto Lawrence and Daniels 2024; Daniels pers. obs). *Peripatopsis lawrencei* is highly variable in dorsal integument colour at Fernkloof Nature Reserve in Hermanus, ranging from rusty orange to brown and slate grey dorsally and can easily be distinguished from *P. fernkloofi* **sp. nov**., since the latter species is royal blue dorsally. In addition, the two species can further be distinguished with the use of leg pair numbers, with *P. lawrencei* possessing 17 + 1 (McDonald et al. 2012), while in *P. fernkloofi* **sp. nov**., 16 leg pairs are present. Exhaustive attempts to recollect the species were unsuccessful. The species appears to be very rare.

***Peripatopsis limietbergi* sp. nov**.,

urn:lsid:zoobank.org:act:31DECE4D‐1957‐499A‐B38B‐08B6D92395AB.

(Figures 1, 2, 3, 4, 5F, 6N–O, 7E–F; Table 2).

Holotype: 1 f, Bainskloof Pass, 33°35′798′′S, 19°07′127′′E, Limietberg, Western Cape province, South Africa, collected 27 June 2022, by R. Basson (SAM‐ENW‐C015310).

Paratype: 2 f and one specimen (not sexed not), Mitchell's Pass, 33°24′44′′S, 19°16′30′′E, Ceres, Western Cape province, South Africa, collected 20 July 2006 by S.R. Daniels (SAM‐ ENW‐C006559).

Additional Material: 1 m and 1 f, Mitchell's Pass, 33°24′44″S, 19°16′30″E, Ceres, Western Cape province, South Africa, collected 17 September 2010 by D.E. McDonald and A. Abels, (SAM‐ENW‐C006511); 2 specimens (not sexed of which one broken in half), Bainskloof Pass, 33°35′798″S, 19°07′127″E, Limietberg, Western Cape province, South Africa, collected 21 June 2006 by S.R. Daniels and H. Van den Worm, (SAM‐ENW‐ C006561).

Description: Holotype 21.3 mm in length, 4.36 mm in width, ranges from 15.96 mm in length to 2.65 mm in width (Table 2).

Colour pattern: Dorsally blue (Figure 5F), ventrally pearl white when alive.

Leg pair number: Males with 18 leg pairs, last leg pair highly reduced with claw in holotype. Leg pairs vary between 16 and 18 (Table 2).

Integument: Dermal papillae concave‐conical with eccentric sensory bristle. Six dorsal and four ventral scale ranks in both Bainskloof Pass and Mitchell's Pass of primary dermal papillae (Figure 6N–O). Elliptical‐shaped chemoreceptors between antennal folds (Figure 7E).

Genital opening: With a distinctive pattern of very narrow, elongated anterior and posterior pads, with a small pair of median pads. Elongate and compressed, with a flattened and expanded apex. Apical slit horizontal or indented in the form of a small notch (Figure 6D, Daniels et al. 2013).

GenBank: *COI*, Mitchell's Pass, EU 855358–EU 855362; Bainskloof Pass, EU 855357 (Daniels et al. 2009); *18S rRNA*, Mitchell's Pass, EU 855541, KC 766090 (Daniels et al. 2013).

Etymology: Named after the blue color of the integument.

Distribution: Bainskloof Pass and Mitchell's Pass in the Limietberg Mountains, Western Cape province, South Africa.

Habitat: Sheltered areas within gorges and along rivers high on mountain slopes. Collected within moss and decaying logs close to waterfalls and streams.

Remarks: *Peripatopsis limietbergi* **sp. nov**., is highly divergent from its sister species, *P. purpureus s.s*. based on DNA sequence data from the *COI* data and *18S rRNA* (Figures 2 and 3). *Peripatopsis purpureus s.s*. is restricted to the type locality, at the terminal end of the Mollenaars Hike, Du Toitskloof Pass, Western Cape province. The two sister species (*P. limietbergi* **sp. nov**., and *P. purpureus s.s*.) can also be differentiated based on dorsal integument colour of live specimens and dorsal and ventral scale counts. The discovery of a ventral organ in *P. limietbergi* is novel (Figure 7F). Both sister species are generally found under carpets of moss in close proximity to streams in small Afrotemperate forest patches. The Bain's Kloof Pass holotype was found in sand under a rock. The species in regionally narrowly distributed and only know from Mitchell's Pass and Bainskloof Pass.

***Peripatopsis jonkershoeki* sp. nov**.,

urn:lsid:zoobank.org:act:01DF5992‐861E‐482B‐82C6‐0F02D2F5F29F.

(Figures 1, 2, 3, 4, 6P–Q; Table 2).

Holotype: 1 specimen (not sexed), Tweede Waterfall hike, 33°56′393″ S, 18°51′344″ E, Jonkershoek Nature Reserve, Western Cape province, South Africa, collected 4 April 2022 by S.R. Daniels (SAM‐ENW‐C015316).

Paratype: 1 specimen (not sexed), same collection information as for holotype (SAM‐ENW‐C015317).

Additional Material: 1 m, Jonkershoek site 1, collected by S.R. Daniels, Jonkershoek Nature Reserve, Western Cape province, South Africa (SAM‐ENW‐C006563).

Description: Holotype measurements: length 14.57 mm, width 2.78 mm (Table 2).

Colour pattern: Slate grey to light purple dorsally, white ventrally.

Leg pair number: 18 (Table 2).

Integument: Primary dermal papillae with eight dorsal and six ventral scale ranks (Figure 6P–Q).

Genital opening: Gonopore star‐shaped.

GenBank: *COI*, Jonkershoek Nature Reserve site 1, EU 855350–EU 855354; EU 855327 (Daniels et al. 2009); PP 550029–PP 550030 (present study); *18S rRNA*, Jonkershoek Nature Reserve site 1, KC 766085 (Daniels et al. 2013).

Etymology: Named after the Jonkershoek Mountains (B) outside Stellenbosch, Western Cape province, South Africa.

Distribution: Narrow endemic.

Habitat: In a small ravine gully, in decaying logs of wood towards the Tweede Waterfall hike, Jonkershoek Nature Reserve in a very small Afrotemperate forest patch with large boulders.

Remarks: In a gorge at Jonkershoek Nature Reserve, *Peripatopsis lawrencei* occurs on the valley floor at lower altitudes (McDonald and Daniels 2012). The latter species is genetically divergent from *P. bolandi s.s*. based on *COI* and *18S rRNA* data.

***Peripatopsis kogelbergi* sp. nov**.,

urn:lsid:zoobank.org:act:FB28BB64‐3866‐40 EB‐B542‐1F8C37ECD8EE.

(Figures 1, 2, 3, 4, 6R–S; Table 2).

Holotype: 1 m, Kogelberg Biosphere Nature Reserve, 34°19′59′′S, 18°57′01′′E, Western Cape province, South Africa collected (no date provided) 2011 by McDonald & Abels (SAM‐ENW‐C015307).

Paratype: 1 m, 2 j, same locality information as above (SAM‐ENW‐C015306).

Description: Holotype measurements: length, 16.73 mm, width 3.71 mm (Table 2).

Colour pattern: Slate black dorsally, white ventrally.

Leg pair number: 18 (Table 2).

Integument: Primary dermal papillae four dorsal scales, and three scales ventral (Figure 6R–S).

Genital opening: Gonopore star‐shaped.

GenBank: *COI*, Kogelberg Biosphere Nature Reserve, EU 855339 (Daniels et al. 2009); KC 766114–KC 766115 (Daniels et al. 2013).

Etymology: Named after Kogelberg Biosphere Nature Reserve, Western Cape province, South Africa.

Distribution: Appears highly endemic.

Habitat: Fragmented Afrotemperate forest patch in a deep kloof (gorge) at Kogelberg Biosphere Reserve (B).

Remarks: Genetically and morphologically distinct from *P. lawrencei* also collected from Kogelberg Biosphere Reserve and sympatric with both *P. bolandi s.s*. and *P. kogelbergi* **sp. nov** (Nieto Lawrence and Daniels 2024).

## Author Contributions

**Savel R. Daniels:** conceptualization (equal), data curation (equal), funding acquisition (equal), investigation (equal), project administration (equal). **Aaron Barnes:** formal analysis (lead), methodology (lead), software (equal), writing – review and editing (equal).

## Conflicts of Interest

The authors declare no conflicts of interest.

## Data Availability

All new DNA sequence data is deposited in GenBank. All DNA sequence data added to additional information (for review not for publication).
